# Perceived roughness of glossy objects: The influence of Fresnel effects and correlated image statistics

**DOI:** 10.1167/jov.21.8.1

**Published:** 2021-08-02

**Authors:** Franz Faul

**Affiliations:** 1 Institut für Psychologie, Universität Kiel, Kiel, Germany

**Keywords:** gloss perception, perceived roughness, material perception

## Abstract

The roughness of a shiny surface determines how sharp the reflected image of the surroundings is, and thus whether the surface appears highly glossy or more or less matte. In a matching experiment, subjects were asked to reproduce the perceived roughness of a given surface (standard) in a comparison stimulus (match), where the standard and the match could differ in both shape and illumination. To compare the effect of the reflection model on the accuracy of the settings, this was done for two different reflectance models (bidirectional reflectance distribution function [BRDF]). The matching errors were smaller, that is, the constancy under shape and illumination changes higher, when Fresnel effects were physically correctly reproduced in the reflectance model (Fresnel-BRDF) than when this was not the case (Ward-BRDF). The subjects’ settings in the experiment can be predicted very well by two image statistics, one of which is based on the mean edge strength and the other on a local discrete cosine transform. In particular, these predictions also reflect the empirically observed advantage of the Fresnel-BRDF. These results show that the constancy of perceived roughness across context changes may depend on the BRDF used, with Fresnel effects playing a significant role. The good prediction of subjects’ settings using the two image statistics suggests that local brightness variance, which affects both image statistics, can be used as a valid cue for surface roughness.

## Introduction

Shiny surfaces play an important role in everyday life and it is therefore of considerable interest how humans recognize such materials and their properties. In visual perception, the processes responsible for this must use regularities in the retinal image of surfaces that are characteristic of this type of material. Because glossy materials can be detected in single photos, it is clear that at least part of the relevant information is already available in static images.

A classical approach to identifying such regularities in the visual input is to consider the physics of image generation. By analyzing appropriate generative models, specific image features or image statistics (“cues”) can be derived that objectively correlate with a particular material property. The hypothesis that an image feature identified in this way actually plays a role in visual perception must then be tested empirically.

This approach is also the focus of the present study. The question is by which image features the perceived roughness of glossy surfaces is determined and how constant this impression is across variations in shape and illumination. This problem has already been investigated in numerous studies, both with local light sources (Honson et al., 2020; Nishida & Shinya, 1998; Wendt & Faul, 2018, 2017; Wendt, Faul, & Mausfeld, 2008) and with more realistic global illuminations (Fleming, Dror, & Adelson, 2003; Marlow & Anderson, 2013). These studies consistently show that the perceived roughness of glossy surfaces depends on the sharpness of the mirror image of the environment (which in simple cases may consist only of light sources) it reflects. It has also been shown that illumination and shape affect perceived roughness, implying that there is limited constancy with changes in shape or illumination.

However, a potential problem with most of the aforementioned studies is that they used in stimulus generation simplified reflectance models such as the Phong model (Phong, 1975) or the Ward model (Ward, 1992), which do not properly account for Fresnel effects that are based on an essential physical law of specular reflection. Roughly speaking, Fresnel effects describe how the spatially varying intensity of the mirror image of the environment on the surface of a shiny object depends on its shape. Neglecting Fresnel effects seems especially problematic for global illumination, where there is an extended, contiguous mirror image that encompasses large areas of the environment.

The current study specifically addresses the question of whether neglecting Fresnel effects impacts the perceived roughness or mattness of a glossy surface and whether this may have led to an underestimation of constancy performance in previous studies. The results in Faul (2019) show that such concerns are not unfounded. There, it was shown that correctly simulated Fresnel effects can significantly affect the quality and strength of the gloss impression and that the constancy of perceived reflectance strength across changes in shape and illumination can also improve.

The present study can be seen as an extension of this previous investigation of perceived reflectance strength to an additional dimension of the gloss impression. The question of whether Fresnel effects also influence the perceived roughness of glossy surfaces is of particular interest because Fresnel effects directly influence only the intensity of the mirror image, but not its sharpness, which according to previous findings is closely related to perceived surface roughness (Cicco, Wijntjes, & Pont, 2019; Kim, Tan, & Chowdhury, 2016; Marlow & Anderson, 2013; Marlow, Kim, & Anderson, 2012).

**Figure 1.**
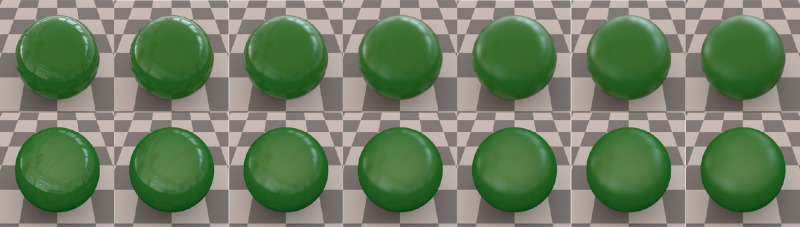
Roughness scale with correct Fresnel effects (top) and for the Ward-BRDF, which does not correctly reproduce Fresnel effects. The roughness parameter α varies from left to right in 7 approximately equally spaced steps from 0.001 to 0.12, that is, in the range used in the experiment for the standard stimuli (bottom).

To explore this question, two matching experiments were conducted that were identical except for the reflectance model used: In one, the stimuli were computed with the Ward model (Ward, 1992), and in the other, with an alternative model that correctly accounts for Fresnel effects (Walter, Marschner, Li, & Torrance, 2007). Figure 1 shows an example of how the gloss impression of the stimuli created with these two reflection models changes with increasing roughness. In each case, subjects had to match the perceived roughness or mattness of the surfaces across different illuminations and shapes. To anticipate, also in this case correctly simulated Fresnel effects had a positive influence both on the gloss impression and on the constancy of the perceived roughness under shape and illumination changes.

In an attempt to identify stimulus features responsible for the roughness settings in the experiment, two different methods were considered, which are based on statistics of local variance and local edge strength. These statistics were found to have a very regular and strictly monotonic relationship with simulated surface roughness. This relationship also proved to be very robust, that is, it hardly depended on diffuse color, shape, and illumination. The fact that this robustness was even more pronounced with correct Fresnel effects than with the Ward model could explain the improved constancy performance found in the experiment under this condition. Moreover, these image statistics predicted the subjects’ settings significantly better for stimuli with correct Fresnel effects than for stimuli rendered with the Ward model.

Before discussing these findings in greater detail, the remainder of the introduction briefly outlines some physical, empirical, and methodological aspects that play an important role in these investigations. A first topic are basic principles of image generation for glossy surfaces and the associated image regularities. In addition, the essential role of the reflectance model in studies of gloss perception is briefly discussed. Finally, problems of the frequently used Ward model and the related consequences for the perceptual impression are described.

### Image generation with glossy surfaces

Gloss perception refers to a material property of surfaces. For visual perception, only the optical properties of materials are of direct interest, that is, in the case of opaque surfaces how they reflect incident light of wavelength λ. Important aspects of the reflectance behavior at a surface point p can be described by the bidirectional reflectance distribution function (BRDF) $f(p,\omega_{i},\omega_{o},\lambda )$, which gives for each incident direction $\omega_{i}$ the spatial distribution of the reflected light over all possible reflection directions $\omega_{o}$ (and vice versa). If, in addition to the BRDF, the shape and illumination of the surface are known, and furthermore the imaging geometry of the optical system used, then its image projected onto a two-dimensional sensor surface can be calculated very accurately according to known physical laws.

The characteristic feature of glossy appearing surfaces is the presence of specular reflection. The basic form of the associated BRDF is well-known. In the special case of “ideal specular” reflection, which occurs with perfectly smooth surfaces, it is very simple: light incident from direction $\omega_{i}$ is reflected in exactly one direction $\omega_{o}$, the direction of reflection, where $\omega_{i}$ and $\omega_{o}$ lie in the same plane and have the same angle to the surface normal. In the more common case of diffuse specular reflection, the direction of the reflected light varies within a cone about the specular direction. For a given direction of incidence, the BRDF then roughly has the shape of a lobe oriented in the direction of reflection. The width of the lobe increases with the roughness of the surface. In so-called microfacet models this roughness is modeled by the variance of the orientation of the normal vectors of “facets,” that is, tiny, ideally reflecting partial surfaces (Cook & Torrance, 1982).

Besides the direction of the specularly reflected light, a second important aspect is the relative fraction of the incident light energy that is reflected in a certain direction. This fraction is described in optics by Fresnel's equations, which exist in two versions, one for metals and the other for nonconductors (dielectrics). Typical dielectrics are partially transparent materials such as glass, liquids, and plastics. In both classes of materials, Fresnel's equations depend on the refractive index and the angle of incidence of the light, but in very different ways. For dielectrics, which are considered exclusively in the following, the reflected fraction is minimal at perpendicular incidence and increases strictly monotonically and nonlinearly up to a value of 100% at the maximum incidence angle of $90^{\circ }$. The refractive index is actually a function of wavelength, but for typical dielectrics it hardly changes in the range of visible light and is therefore often approximated by a single constant value. The minimum reflectance at perpendicular incidence increases with the refractive index. However, the associated change in global reflectance is of less practical significance than the directional dependence of reflectance strength (called “Fresnel effects” in the remainder), because the refractive index is usually constant for a given material and lies in a narrow range of about 1.3 to 2.0 for typical dielectrics.

Many glossy surfaces can be described as a combination of a base material, for example, a diffuse-reflective surface such as wood, with a transparent coating, such as a clear varnish (Jakob, d'Eon, Jakob, & Marschner, 2014; Weidlich & Wilkie, 2007). In this common special case, which we consider exclusively in this article, the BRDF of the layered material is, to a first approximation, a combination of the BRDF of diffuse reflection described by a hemisphere for each direction of incidence and the lobe-shaped BRDF of the diffuse specular reflection.

### Correlated image properties and gloss cues

From these basic properties of the BRDF of shiny opaque surfaces, essential properties of the associated retinal image can be derived: On the transparent layer of the surface of such objects, an observer sees a mirror image of the surroundings, which is distorted and (owing to Fresnel effects) varies in intensity depending on the shape of the surface. In addition, as the roughness of the surface increases, the mirror image becomes blurrier and lower in contrast. Through the transparent layer on which the mirror image is formed, one sees the diffuse surface, whose color can be homogeneous or spatially varied (textured).

When recognizing a surface of this type as glossy, the challenge is first to decide whether the pattern in the retinal image should be interpreted as a texture of a diffuse surface or as a reflection of the surrounding (Fleming et al., 2003). If specular reflection is assumed, then the intensity and sharpness of the mirror image must be determined, from which the strength of the mirror reflection and the roughness of the surface can then be inferred, which together determine the gloss impression. For this purpose, it is necessary to separate the contributions of diffuse and specular reflection. This decomposition of the retinal image into causal components is particularly difficult in static, monocularly viewed stimuli and seems solvable only if statistical regularities of the external world are taken into account (Barron & Malik, 2016).

In studies of gloss perception, many such regularities have been identified. In simple scenes with rather local illumination, highlights, that is, the reflection of local light sources, play an essential role (Beck & Prazdny, 1981; Berzhanskaya, Swaminathan, Beck, & Mingolla, 2005). The available evidence suggests that particularly bright regions in the image are interpreted as highlights only if they are compatible in shape, orientation, location, and color with the detected surface shape and illumination (Nishida et al., 2008; Todd, Norman, & Mingolla, 2004). If a gloss impression is triggered, the blurring of the edges of the highlights seems to be the main cue for the roughness of the material.

Recent work increasingly refers to the more realistic case of global illumination, where direct illumination by isolated light sources is combined with indirect illumination by light reflected from neighboring surfaces. Global illuminations can be approximately simulated by so-called image-based lighting, which uses illumination maps with high dynamic range (Debevec, 2008). In such cases, the spatial distribution of illumination, that is, the structure of an illumination map, also plays a major role (Adams, Kucukoglu, Landy, & Mantiuk, 2018; Fleming et al., 2003; Olkkonen & Brainard, 2011; Zhang, de Ridder, & Pont, 2018; Zhang, de Ridder, Barla, & Pont, 2019). The findings of Fleming et al. (2003) suggest that the identification of a reflectance pattern as illumination is more successful when the illumination maps show real environments. Object shape also seems to play a role (Olkkonen & Brainard, 2011; Vangorp, Laurijssen, & Dutré, 2007). This is plausible because the mirror image of the surroundings is distorted in a characteristic way depending on the shape of the object. Also in this more general case, the gloss impression depends on the properties of bright highlights and in particular also their congruence with shape information (Kim, Marlow, & Anderson, 2011; Marlow & Anderson, 2013; Marlow, Kim, & Anderson, 2011). Of particular relevance to the current investigation are the findings in Faul (2019), which suggest that Fresnel effects, that is, the correspondence between object shape and the intensity of the mirror image, may also play a role in gloss perception.

### The role of the reflection models

The reflection behavior of real objects is usually very complex and can only be captured in detail by explicit measurement (Dupuy & Jakob, 2019). For practical purposes, however, parameterized reflectance models often provide a sufficient approximation. They also have the advantage that the surface properties can be varied within wide limits by adjusting a few parameter values. Reflection models generally represent approximations. Which reflection model, or approximation, is best depends on the application as well. In general, it can be said that the more complete and accurate physical regularities are simulated, the more complex the calculation of the model becomes. Therefore, in real-time applications, one usually chooses coarser approximations than in applications where scenes can be rendered without tight time constraints.

The question of a suitable reflection model is also of great relevance for perceptual research, because artificially rendered objects and surfaces are increasingly used as stimuli in experiments. A major reason for this is the associated flexibility in stimulus generation, which includes in particular the possibility to isolate certain regularities and to produce physically incorrect stimuli. However, this practice carries the risk that a potential influence of certain physical regularities on perceptual performance can principally not be detected if they are not correctly realized in the models used. As a consequence, misleading statements about the performance of the visual system, in particular about the constancy of the perceptual impression under variation of the context, may result.

In recent years, research on gloss perception has frequently used Ward's (1992) reflection model. On the one hand, this choice has pragmatic reasons, because it is a relatively simple model; on the other hand, comparability with previous work and the existence of a psychophysical reparametrization of the model (Cheeseman, Ferwerda, Maile, & Fleming, 2021; Pellacini, Ferwerda, & Greenberg, 2000) also seem to play a certain role.

This practice is potentially problematic because the original Ward model has two major drawbacks that have been known for some time: First, it is not energy conserving, and second, it does not correctly account for Fresnel effects (Ngan, Durand, & Matusik, 2005). For rough surfaces, it also ignores a geometric attenuation factor, but for typical surface parameters this has only a very minor effect (see Appendix A.2). Although the problem of the lack of energy conservation was solved by a modification of the model (Geisler-Moroder & Dür, 2010), the second problem of ignoring Fresnel effects was not. It manifests itself in the fact that the intensity of the mirror image does not change in a correct way with the incidence angle of the light, but is comparatively too large with perpendicular incidence and too small with oblique incidence.

In Faul (2019), some consequences of missing or incorrect Fresnel effects on gloss perception have already been investigated. For this purpose, the Ward-BRDF was compared with an alternative Fresnel-BRDF (Walter et al., 2007) that correctly simulates Fresnel effects. The main findings of this study were 1) the gloss impression produced with the Fresnel-BRDF was generally qualitatively different from that produced with the Ward-BRDF (see also Guarnera et al., 2020). 2) All other things being equal, the gloss impression tends to be stronger and more realistic with the Fresnel-BRDF. 3) Especially with relatively homogeneous illuminations, the gloss impression was often still largely preserved with the Fresnel-BRDF where it had already completely disappeared with the Ward-BRDF. 4) Perceived reflection strength varied less with shape and illumination changes with the Fresnel-BRDF than with the Ward-BRDF, that is, constancy performance was higher with the Fresnel-BRDF. As an informal observation, it was also found that a “stronger shape impression” is often evoked with a Fresnel-BRDF. For example, a sphere rendered with the Ward-BRDF seems to be flattened compared with a rendering with the Fresnel-BRDF.

These numerous, very distinct differences in perceptual impression, some of which are also evident in Figure 1, suggest that Fresnel effects are taken into account by the visual system in material and shape perception. However, this does not have to be true in principle and for all aspects of the gloss impression, but it has to be tested empirically for each aspect whether and under which conditions a marked effect can be observed.

## Experiment: Constancy of perceived roughness

The aim of the experiment was to determine the influence of Fresnel effects on the perceived roughness of glossy surfaces and to measure the degree of constancy of the corresponding perceptual impression under variation of shape and illumination. For this purpose, the subjects were presented with a fixed object (standard) and a comparison object with adjustable roughness (match), whereby standard and match could differ in shape and/or illumination. The subjects were asked to rate the strength of the gloss impression in the standard on a scale from 0 to 10 and to reproduce the perceived roughness of the standard in the comparison object by making a suitable adjustment. To manipulate the crucial variable “availability of Fresnel effects,” the original Ward-BRDF (Ward, 1992) on the one hand and a BRDF that correctly simulates Fresnel effects (Walter et al., 2007) on the other hand were used as BRDF. The matching was always between stimuli with the same BRDF.

Aspects of this question have already been investigated by Fleming et al. (2003), who determined the constancy of perceived roughness across 13 different illuminations in a similar matching experiment. However, they used only spherical objects and the Ward-BRDF. The match always had the same global illumination, which was different from all illuminations used in the standard. The main result of this study was that the adjustment error was considerably smaller for real-world illuminations than for artificial illuminations.

The study by Faul (2019) measured perceived reflectance of glossy surfaces instead of their perceived roughness, but otherwise had a very similar design, with BRDF, object shape, and illumination as independent variables. In this case, it was found that the constancy performance across different shapes and especially across different illuminations was higher for a Fresnel-BRDF than for a Ward-BRDF. Thus, the current experiment also serves to test whether this finding can be generalized to perceived roughness.

### Methods

**Figure 2.**
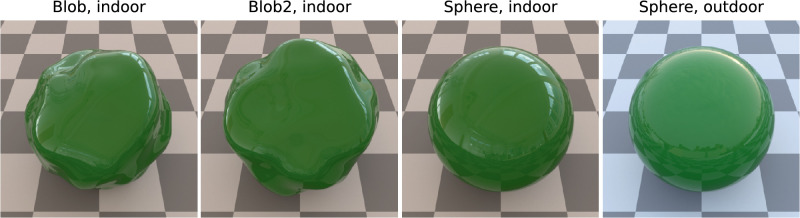
Example stimuli. Three shapes “blob”, “blob2”, and “sphere” were used, as well as an “indoor” and an “outdoor” illumination. All stimuli shown are rendered with Fresnel-BRDF and lowest roughness.

The objects used were a sphere and a “blob.” The “blob” was shown in two views, with the second view, hereafter referred to as “blob2,” rotated 90° about the vertical axis relative to the first. The same illumination maps as in Faul (2019) were used, namely DH206 and DH209 from Dosch Design's Extreme Highres series, showing an “indoor” and an “outdoor” scene, respectively. Some example stimuli can be seen in Figure 2. The stimuli were rendered using Mitsuba (Jakob, 2010), choosing either “ward” or “roughplastic” as the BRDF. Because all shapes and illuminations were used in both standard and match, this resulted in a total of 72 = 9 (shape pairs) × 4 (illumination pairs) × 2 (BRDF) different experimental conditions.

**Figure 3.**
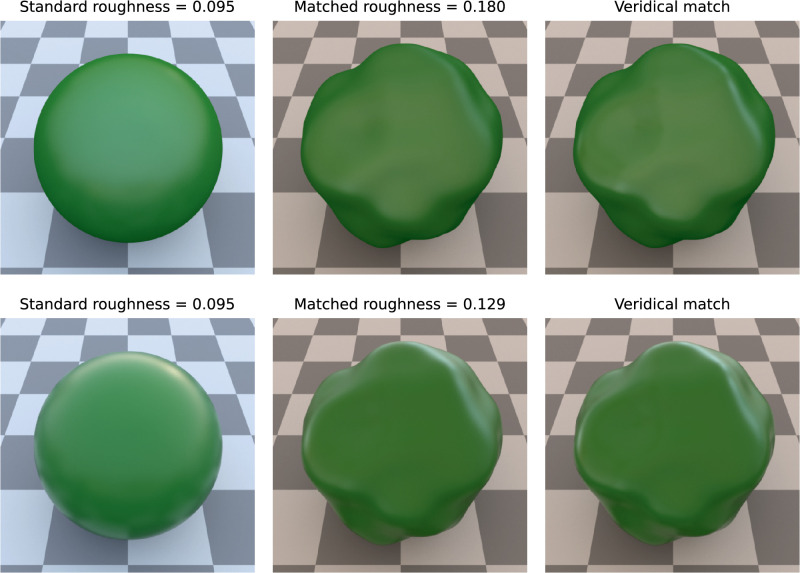
Example settings for Ward-BRDF (top) and Fresnel-BRDF (bottom) in the same condition. From left to right: Standard stimulus, mean setting of the subjects, correct setting.

In both BRDFs, the same values (r = 0.036, g = 0.133, b = 0.022) were used for the diffuse reflection, which lead to a dark green color impression under neutral illumination. To define the intensity of specular reflection, the refractive index for the Fresnel-BRDF was set to ior=1.5. The corresponding parameter $\rho_{s}=0.085$ of the Ward-BRDF was determined via the conversion $\rho_{s}=\left(ior-1\right)0.17$, which has already been used in Faul (2019) to produce for this type of object very similar perceived intensities of the mirror image with both BRDFs.

The roughness parameter α of both BRDFs was varied in 60 equidistant steps from 0.001 to 0.200. This covers the range from highly glossy to very matte (Pellacini et al., 2000). In the Fresnel-BRDF α refers to the distribution of normal vectors in a microfacet model. Here, the Beckmann distribution (Beckmann & Spizzichino, 1987) was chosen to increase the comparability to the Ward-BRDF, in which this distribution is also implemented, albeit in simplified form. In the realized range of values, increasing α leads to a very similar and approximately linear (Pellacini et al., 2000) decrease of perceived roughness in both BRDFs (Figure 1). As noted elsewhere in this article, for rough surfaces the Ward-BRDF neglects not only Fresnel effects, but also a geometric attenuation factor G. In the present situation, however, the effect of G is negligible (see Appendix A.2).

For each of the 36 shape and illumination combinations per BRDF, four values were randomly selected for the standard stimulus from the 60 roughness levels. The resulting values ranged from 0.014 to 0.122. This restriction on the range of standard values ensured that the subjects’ settings could in principle deviate upward and downward from the target values. This random selection of standard roughnesses was chosen to cover the roughness range as evenly as possible and to make it more difficult to use undesirable adjustment strategies, which are possible with only a few different standard roughnesses. To maintain complete comparability of the two BRDFs, which is central to the research question, exactly the same standard roughnesses were used in both BRDFs. This resulted in a total of 288 settings for each subject. However, because 9 identical values were drawn in the random selection of the 144 standard roughnesses, only 270 different conditions exist.

Objects were presented resting on a checkerboard floor, with r,g,b=0.2 and r,g,b=0.4 chosen as the reflectances of the two gray subsurfaces. The stimuli were computed in high dynamic range format with the bidirectional renderer of Mitsuba (“bdpt”) at a size of 600 × 600 pixels and a sample count of 512 per pixel. From these, low dynamic range images for display were created using the tone mapping method of Reinhard and Devlin (2005), with image-dependent parameters determined in one standard stimulus and held constant for all other stimuli.

Stimuli were displayed on a 24-inch LCD monitor with 1980 × 1200 pixels (Dell P242) against a dark gray background and viewed from a distance of about 60 cm. The room was largely darkened. The two stimuli were shown side by side in the center of the monitor, with the left stimulus always being the fixed standard stimulus. In the right stimulus, the subject could adjust the roughness using the arrow keys of a normal keyboard. The task was to make the perceived roughness in both stimuli as similar as possible. Actually, the subject selected the best-fitting image from 60 pre-rendered images of the corresponding match condition. Since the same 60 roughness values were used in all conditions, an exact match of the nominal roughness value between standard and match was always possible.

During a single session lasting approximately 120 minutes, the 288 stimuli were presented in random order (which was identical for all subjects). In each trial, the first task was to match the perceived roughness of the two stimuli. The participants were instructed to ignore shape and illumination differences as much as possible and to focus solely on the perceived mattness of the materials. Subsequently, the gloss impression in the standard stimulus was to be rated on a scale from 0 (not glossy at all) to 10 (maximum glossy). It was explicitly pointed out in the instructions that the gloss impression can vary on a continuum from matte to highly glossy.

Eleven subjects participated in the experiment (6 female, 5 male; average age 26 years). Participation was voluntary and without compensation. All of them had normal or corrected-to-normal vision. Except for one person who also acted as the experimental supervisor, all subjects were naive to the question of the experiment, presumably did not know the relationship between surface roughness and perceived mattness, and especially were not told that different reflection models were used in the experiment. The data from one participant, who in the follow-up interview reported problems perceiving glossy objects as such and who also produced settings that were significantly different from those of the other participants, were excluded from the data analysis. The inclusion of these data would not fundamentally change the overall results.

### Results

**Figure 4.**
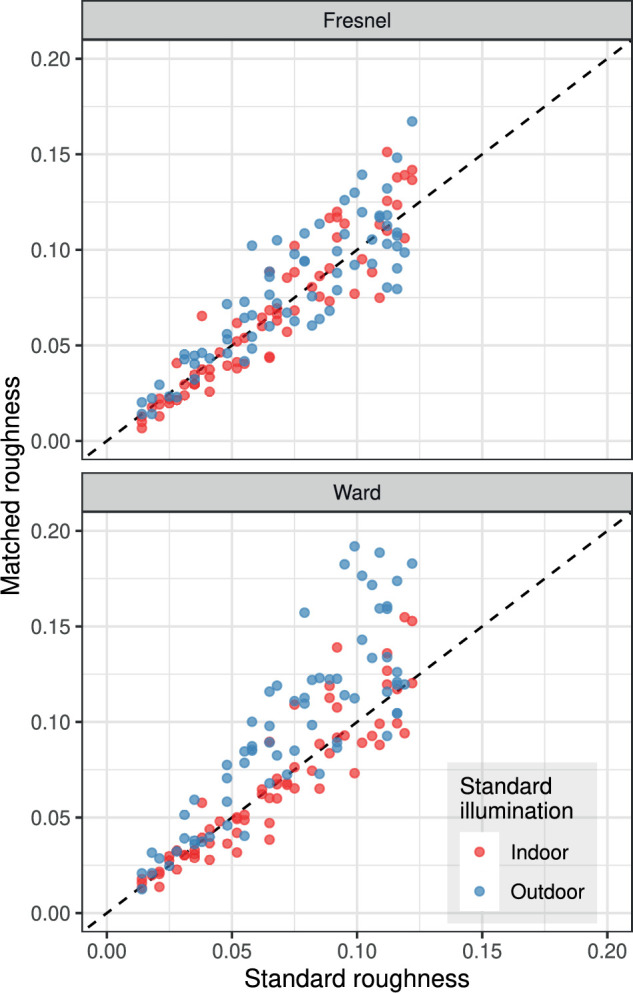
Roughness settings in the 135 different conditions for each of the two BRDFs, averaged over the 10 subjects. Data are grouped by illumination type in the standard stimulus.

Figure 3 shows exemplary matching results for both BRDF. Figure 4 presents the mean settings in all 270 different conditions (in 18 cases, 2 identical conditions were combined, doubling the sample size there). Overall, there is a relatively good agreement between the given standard values and the settings, which is higher for the Fresnel-BRDF ($R^{2}=0.631$ for 1440 individual settings and $R^{2}=0.813$ for 135 mean values) than for the Ward-BRDF ($R^{2}=0.562$ and 0.734, respectively). This difference is significant (p<0.001 and p=0.0102, respectively, for one-sided tests).

The matching errors correspond with the vertical deviation from the diagonal line. They tend to increase with increasing roughness of the standard. For the Fresnel-BRDF, the errors are fairly symmetrically distributed, while for the Ward-BRDF, the distribution is much more asymmetric, especially with outliers in the direction of too large settings. Grouping by standard illumination reveals that these outliers occur primarily when the relatively homogeneous “outdoor” illumination is present in the standard stimulus.

**Figure 5.**
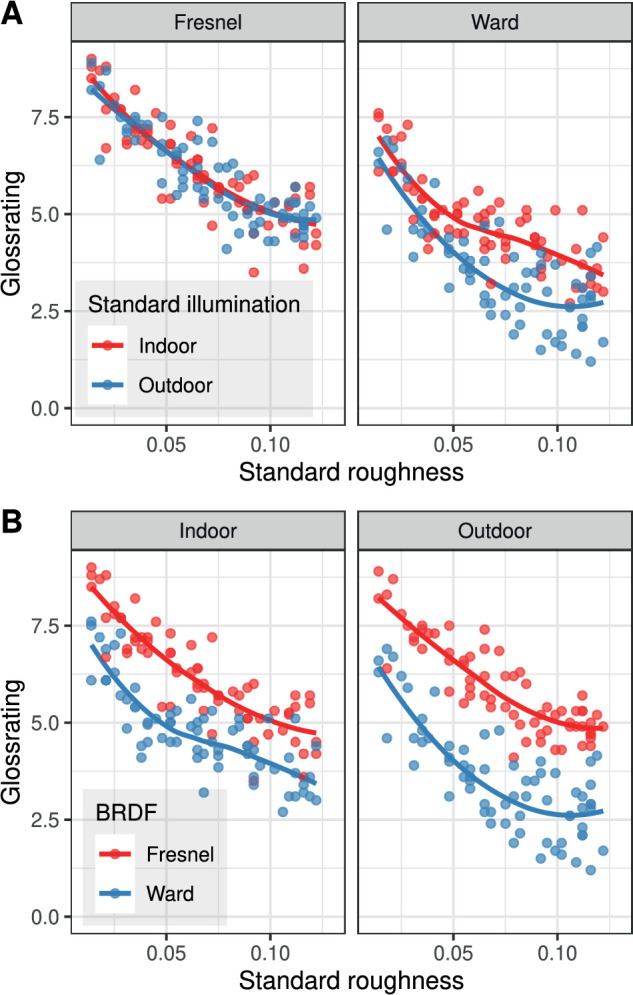
Two views of the mean gloss ratings of the standard stimulus as a function of roughness. (A) Comparison of the ratings as a function of the standard illumination for both BRDFs. (B) Comparison of the ratings as a function of the two BRDFs for both standard illuminations.

Figure 5 shows the mean gloss ratings of the standard stimulus as a function of roughness and a smoothing curve through the data points (Loess with span = 1). The general and expected trend is that the gloss impression decreases with increasing roughness. Figure 5A indicates that the relationship between gloss impression and roughness is nearly linear for the Fresnel-BRDF, and that the type of standard illumination has virtually no systematic effect on the gloss impression. The analogous relationships for the Ward-BRDF are less clear and change systematically with the standard illumination, with the gloss impression being weaker with the outdoor illumination. Figure 5B provides a direct comparison between the BRDFs for each standard illumination. The results confirm the finding in Faul (2019) that the gloss impression is significantly decreased with the Ward-BRDF, with the difference being particularly large for relatively homogeneous illuminations.

**Figure 6.**
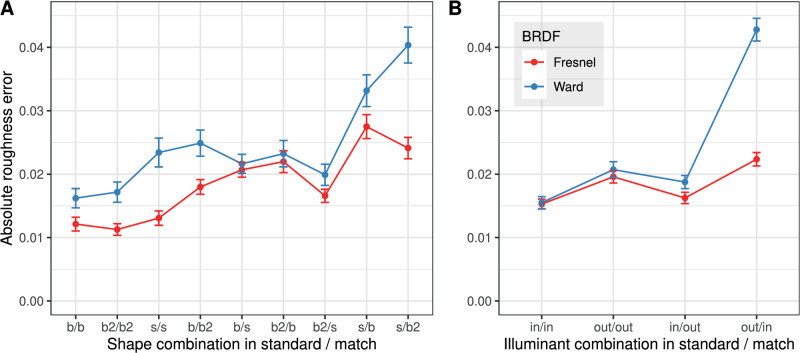
Mean absolute error of the roughness setting (±1 SEM) as a function of (A) shape combination and (B) illumination combination in standard and match for both BRDFs. The sample size per data point is n=160 in A, and n=360 in B. In the axis labels, “b,” “b2,” and “s” stand for “blob,” “blob2,” and “sphere,” respectively, and “in” and “out” stand for “indoor” and “outdoor” illumination. Note: In these and subsequent plots, values at the levels of a discrete UV are connected to facilitate the comparison.

Figure 6 plots the mean absolute errors of the roughness settings as a function of the shape and the illumination combination in standard and match. In this analysis, it is important to note that the four roughness levels in the 36 shape/illumination combinations realized in the experiment were drawn randomly and independently, and thus are in general not identical. Because the matching error increases systematically with the roughness in the standard stimulus (cf. Figure 4), comparisons between individual levels of the shape and illumination factors are problematic (comparisons between BRDFs are not affected). To minimize this sampling effect, multiple conditions are combined in Figures 6A and 6B so that each data point represents an average over 16 and 36 different levels of roughness, respectively.

Figure 6B shows the influence of the illumination combination in standard and match on the errors. As a general trend under all illumination combinations, it can be observed that the errors are smaller with the Fresnel-BRDF than with the Ward- BRDF. However, greater differences between the BRDFs only occur when different illuminations are used in standard and match (“in/out” and “out/in”). An interesting asymmetry is also revealed: the error in the Ward-BRDF and the difference from the Fresnel-BRDF is much greater in the “out/in” condition, that is, when the more homogeneous “outdoor” illumination is used in the constant standard stimulus rather than in the variable match stimulus.

Figure 6A shows the analogous analysis for all shape combinations in standard and match and a similar picture emerges: In all shape combinations, the error is smaller with the Fresnel-BRDF than with Ward-BRDF. For both BRDFs, the error seems to be particularly large when the standard stimulus is a sphere. With the Ward-BRDF this is true even if the standard and the match have the same shape (“s/s”). However, it should be noted here that a noticeable shape effect occurred only in the illumination condition (“out/in”), so that the sampling problem mentioned above complicates the interpretation of the differences between specific shape combinations.

**Figure 7.**
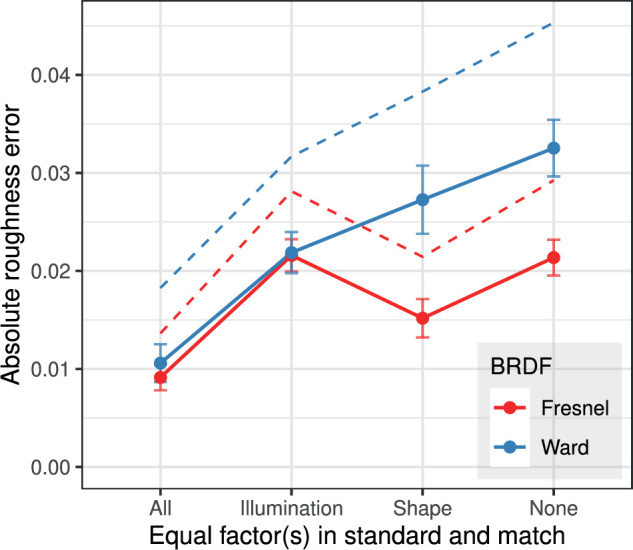
Mean absolute error of the roughness settings (± 2 SEM) as a function of the degree of agreement between standard and match with respect to shape and illumination. To facilitate direct comparisons with previous results of Fleming et al. (2003), also the corresponding RMS errors, where large deviations enter with greater weight than small ones, are given as dashed lines.

An alternative view of how the errors depend on shape and illumination differences in standard and match is shown in Figure 7. Here, the data are grouped according to the correspondence of the respective factor in standard and match. In each case, the relevant reference is the condition shown on the left (“All”), where the subjects performed a symmetric match. It can be seen that with the Ward-BRDF, a difference in illumination (“shape equal”) has a significantly stronger effect on the error than a difference in shape (“illumination equal”).

**Figure 8.**
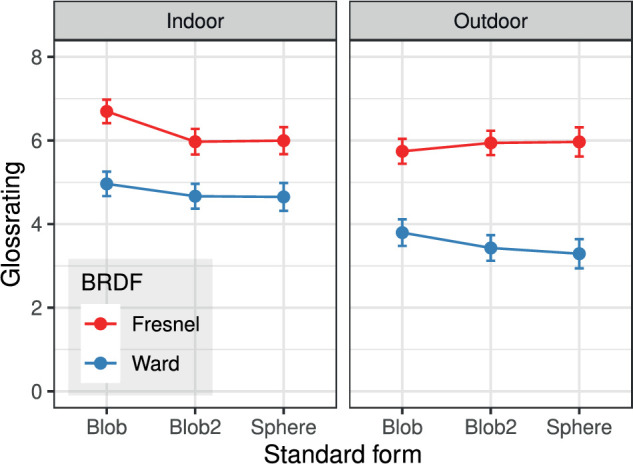
Mean gloss ratings (±2 SEM) for the standard stimulus as a function of shape and illumination (n=240 per data point).

The dependence of the gloss rating of the standard stimulus on shape and illumination is shown in Figure 8. With the Fresnel-BRDF, no systematic effects of these two variables are apparent. With the Ward-BRDF, the shape effect is also very small. However, there is a clear effect of the illumination; as shown in Figure 5, when using the Ward-BRDF, objects seem to be significantly less glossy under the “outdoor” illumination than under the “indoor” illumination.

### Discussion

Overall, these data suggest that, when surface roughness is varied, it can make a substantial difference for both the strength of the gloss impression and the constancy of the roughness impression under shape and illumination changes whether or not Fresnel effects are correctly simulated in the BRDF used in stimulus generation.

#### Perceived gloss

Stimuli rendered with the Ward-BRDF generally received significantly lower gloss ratings than stimuli rendered with the Fresnel-BRDF under the same conditions (cf. Figures 5 and 8). A more detailed analysis revealed that this was true for all subjects, although to different extents. This result is consistent with the findings in Faul (2019).

The current data provide additional information on the nature of the relationship between simulated roughness and perceived glossiness (see Figure 5A). With the Fresnel-BRDF, this relationship is very regular, nearly linear, and seems hardly affected by changes of surface shape and illumination. Thus, with correctly simulated Fresnel effects, the roughness parameter α seems to be a robust predictor of the perceived roughness of a surface. With the Ward-BRDF, the relationship between roughness parameter and gloss rating is not fundamentally different, but it is recognizably more irregular, and the illumination has a much stronger influence: All other things being equal, the gloss impression is clearly weaker with a homogeneous illumination than with a more structured one.

#### Matching errors

The errors of the roughness settings made in the symmetric match, that is, with identical shape and illumination in standard and match, define a lower bound for the error under optimal conditions and are due, for example, to threshold effects. An increase of the error made in the asymmetric matches beyond this baseline value is indicative of a limited constancy of the perceived roughness across shape and illumination changes.

Thus, the results in Figure 7 show that the constancy of the perceived roughness across illumination and shape changes is limited for both BRDFs, but to a much higher degree for the Ward-BRDF. This is particularly true for constancy under illumination changes. When shape *and* illumination are different, the absolute error for the Ward-BRDF increases by more than a factor of three compared to symmetric matching (condition “None”).

Figure 6B shows a clear asymmetry in the case of different illuminations in standard and match: the mean error is significantly larger when the more homogeneous “outdoor” illumination is used in the standard stimulus and the more structured “indoor” illumination in the match stimulus than in the reverse case. This finding is surprising because equality is a symmetric concept and one would therefore expect errors of opposite sign but similar magnitude when swapping the illuminations in standard and match.

This pronounced asymmetry could be explained by the assumption that the interactive variation in roughness during adjustment, which allows comparisons with “neighboring stimuli” and a coarse localization of a stimulus in the overall scale, can be used to mitigate uncertainties about surface roughness that occur with the reduced information in the mirror image of a single static stimulus. The observed asymmetry in the Ward-BRDF would result if one additionally assumes that in an isolated static stimulus a good roughness estimation is possible with the more informative “indoor” illumination, but not with the “outdoor” illumination. If the “outdoor” illumination is used in the invariant standard stimulus, already the *target roughness* for the matching cannot be determined correctly. If, on the other hand, the “indoor” illumination is used in the standard stimulus, then the target roughness is clearly defined. The additional dynamic information when adjusting the match stimulus can then be used to achieve a good match even with the relatively uninformative “outdoor” illumination.

This would mean that the real uncertainty regarding surface roughness given a single static image is underestimated when an interactive adjustment procedure is used. The differences between Ward and Fresnel BRDF should then become even more apparent if a method without these adjustment dynamics is used, such as a staircase procedure.

#### Comparison with previous results

In the aforementioned experiment by Fleming et al. (2003) on the effect of illumination on gloss perception, the subjects were asked to match the perceived strength of specular reflection and the perceived roughness of two glossy spheres across different illuminations. The illumination of the variable match stimulus was always the same and chosen such that it evoked a vivid gloss impression. In each trial, one of 13 illuminations was used in the standard. These included real-world illuminations as well as “artificial” ones that contained few local light sources or a random pattern. The roughness parameter α in the Ward model was changed from 0 to 0.10 in 10 steps of 0.01. Roughness adjustments were made for 10 levels of reflectance. The results indicated that roughness could be matched much more accurately than reflection strength. The root mean square (RMS) errors of the roughness settings were very similar across all real-world illuminations and ranged from about 0.013 to 0.018. For most of the artificial illuminations, the error was significantly increased (on average it was about 0.025).

In the analyses of the current data, the mean absolute error was taken as the relevant error measure because it is unclear why large deviations should be weighted more strongly in the averaging process. However, for a better comparison with the values of Fleming et al., the RMS errors are also included in Figure 7. In the present experiment, the RMS error with the Ward-BRDF is already about 0.018 for symmetric matches, that is, it is of similar size as the errors for the asymmetric matches in Fleming et al. The errors for the asymmetric matches in the current experiment are significantly larger. The relevant comparison value for the same-shape condition is 0.038, which is even larger than that observed for artificial illuminations in Fleming et al.

There are several possible reasons why significantly lower errors were observed in Fleming et al. 1) The maximum standard roughness in Fleming et al. was lower (0.10 vs. 0.12). This may have a large effect on the mean RMS error, since the magnitude of the matching errors systematically increases with the magnitude of the standard roughness (cf. Figure 4) and the RMS statistic weights large errors more strongly. 2) In Fleming et al., the standard values and possible settings for α were restricted to the same range of values from 0 to 0.1, so that for small standard values possible downward deviations were narrowly limited and for large standard values possible upward deviations. The latter, in particular, can lead to an overestimation of the setting accuracy. This can be easily seen from Figure 4: If the possible settings in the present experiment had been limited to a maximum value of 0.1 for default values up to 0.1, especially the largest errors would be significantly decreased. This holds in particular for the Ward-BRDF. 3) Fleming et al. used only 11 levels of roughness, that is, adjustment steps of 0.01. As can be seen in Figure 7, this value corresponds approximately with the mean absolute error found for symmetric matches in the present experiment. Because the setting accuracy decreases significantly with increasing size of the standard roughness, this step size seems somewhat too large for small roughness values. Because errors smaller than the step size cannot be detected, this may also have contributed to a reduction in the observed errors. 4) Three of the four subjects in Fleming et al. were experienced observers, whereas in the present experiment 9 of 10 subjects had little experience with psychophysical experiments.

The problems mentioned in points 2 and 3 seem to be particularly relevant, because together they can lead to a significant underestimation of the error for relatively large *and* for very small standard roughnesses. It is obvious that limiting the maximum setting in the current study to values equal or smaller than the largest standard roughness, would have prevented a realistic estimate of the degree of constancy. There are additional differences between the experiments besides the ones mentioned above that may have an influence, but it is not clear in which direction they affect the setting accuracy.

In any case, the current results confirm the main finding in Fleming et al. (2003) that the type of illumination can have a strong influence on the perceived roughness of a surface. A correct estimation of roughness seems to be especially difficult for homogeneous illuminations, even if they are real-world illuminations. More important than the realism of the mirrored scene seems to be the statistical nature of the illumination map, in particular the number and distribution of light sources (Dror, Willsky, & Adelson, 2004; Pont & te Pas, 2006; Zhang et al., 2019, Zhang, de Ridder, Barla, & Pont, 2020).

**Figure 9.**
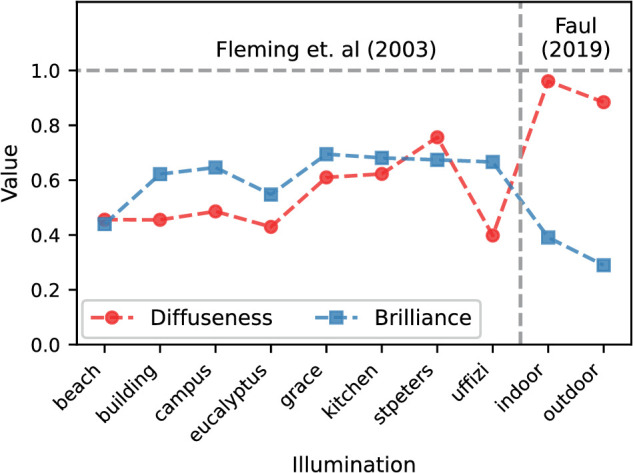
Values of diffuseness and brilliance metrics proposed by Zhang et al. (2019) for the real-world illuminations used in Fleming et al. (2003) and in Faul (2019).

Zhang et al. (2019) distinguish in the direction of decreasing homogeneity the three canonical illumination categories *ambient*, *focus*, and *brilliance*, and found in their Experiment 2 that the gloss rating of a highly glossy material decreased with increasing illumination homogeneity. To objectify these categories, they propose a diffuseness (D) and a brilliance (B) metric. Both are determined from the coefficients of the spherical harmonics of the illumination map and lie between 0 and 1. According to this approach, *ambient* should be characterized by the combination $D^{+}/B^{-}$, *focus* by $D^{-}/B^{-}$ and *brilliance* by $D^{-}/B^{+}$ (the superscripts + and − stand for high and low levels of the metric, respectively). Figure 9 shows the values of these two metrics for the real-world illuminations used in Fleming et al. (2003), as well as for those used in Faul (2019) and in the current experiment. The values for the two illuminations employed here differ significantly from those used in Fleming et al., but are very similar to each other. Both can best be classified as ambient according to the aforementioned criteria. Given this similarity, the large differences in the experimental findings obtained with these two illuminations in the condition with Ward-BRDF are unexpected. A possible interpretation of this result is that the two metrics, which are based on the illumination map alone, ignore some aspects of the illumination that are essential for gloss perception and gloss constancy. For example, a plausible additional factor that is ignored in these metrics is that the relevant portions of the illumination map depend on the orientation of the illumination, the object, and the observer relative to each other (Zhang et al., 2020).

Because divergent results with the two illuminations occurred primarily with the Ward-BRDF, this unexpected result could alternatively be attributed to deficiencies in this BRDF, particular the lack of correct Fresnel effects. With regard to gloss perception in everyday life, deviations from gloss constancy owing to shortcomings in the reflection model are to be regarded as artifacts. When interpreting experimental findings, these should be clearly separated from genuine limitations of the perceptual apparatus.

Our results also show (cf. Figures 6 and 7) that shape differences can also affect the setting accuracy, with the strength of the effect also depending on the illumination. Although these results do not allow definitive conclusions to be drawn, there is a trend that the robustness of the roughness estimate increases with the number of different curvatures.

## Prediction of roughness settings from image features

In Experiment 1, systematic effects of the BRDF on gloss perception and gloss constancy were found. With correct Fresnel effects, the roughness settings were on average more accurate and, in particular, more robust against shape and illumination changes. The aim of the following investigation is to identify image regularities that allow an approximate prediction of the subjects’ adjustment behavior. Image features with these properties could then be regarded as possible cues for perceived roughness.

**Figure 10.**
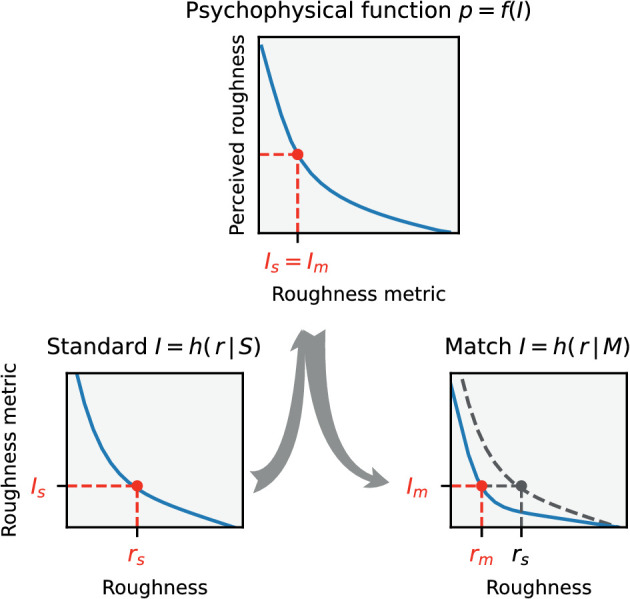
Assumptions about the process underlying an asymmetric match of perceived roughness. Bottom: Standard and match conditions S and M, given by shape and illumination, influence the dependence of an image statistic (“roughness metric” I) on surface roughness r: I=h(r|S) and I=h(r|M), respectively. Top: A psychometric function p=f(I) assigns a perceived roughness p to each value of the roughness metric. The subject chooses the roughness $r_{m}$ in the match such that the perceived roughness is the same in both stimuli, that is, $I_{s}=I_{m}$ holds. In the diagram on the bottom right, h(r|S) is additionally drawn as a gray dashed line. From this diagram it can be seen that $r_{m}$ generally deviates from $r_{s}$ if h(r|M) and h(r|S) do not exactly coincide. The form of the function f, in contrast, has no influence on the result of the matching.

Figure 10 illustrates the assumptions made about the factors involved in the asymmetric matching process. It is assumed that when matching the standard stimulus $R_{s}$ with n possible match stimuli $R_{m}^{i}$, subjects (primarily) use an image statistic I that has a regular relationship I=h(r) to the roughness r of the depicted surface and can thus be used as a roughness metric. However, the exact relationship may additionally depend on the context, in the present case the illumination and the shape of the object. This is indicated by the notation h(r|S) and h(r|M), respectively, where S and M stand for the context condition in standard and match, respectively. It is further assumed that the image statistic I determines the perceived roughness via a psychometric function f(I). The subject chooses the roughness $r_{m}$ in the match where $f\left(I_{s}\right)=f\left(I_{m}\right)$ holds. Because f is assumed to be strictly monotonic, it follows that $I_{s}=I_{m}$. This shows that the exact form of the function f does not affect the matching. Figure 10 illustrates that systematic errors $r_{m}-r_{s}$ of the roughness settings occur only when h(r|S) and h(r|M) differ.

The roughness metric I we are looking for should ideally have the following general properties. 1) It should be objectively determinable from a single static stimulus and the operations required to do so should be neurologically plausible. 2) It should have a strictly monotonic functional relationship to the simulated roughness, that is, to the roughness parameter α of the BRDF. 3) This relationship should be robust, that is, be affected as little as possible by changes in shape and illumination.

With respect to the present investigation, it is further required that the subjects’ settings can be correctly predicted by the metric. This includes that a) the prediction is close to the mean settings of the subjects, and, closely related, that b) the deviations of the predictions from the true values follow the pattern observed in the experiment and, moreover, are smaller for the Fresnel BRDF than for the Ward BRDF.

### Potential roughness metrics

The situation realized in the experiment is particularly well-suited for this investigation because, in the static images used within a condition, the roughness of the surface was varied in isolation over a wide range of values. The correlates of this manipulation in the image are essentially the sharpness and contrast of the mirror image, both of which decrease monotonically with increasing roughness. A slight difficulty arises from the fact that the specular image is not isolated, but superimposed on the diffuse color. In the current investigation, however, we have a particularly simple case in this respect, in that the diffuse reflection is homogeneous.

The task of the subjects in the experiment is similar to the goal of an image-based autofocus procedure implemented in cameras or microscopes, where within an ordered “stack of out-of-focus images” created by shifting the lens, the sharpest image is searched for and the corresponding lens position is selected (Groen, Young, & Ligthart, 1985). However, a significant difference is that, in the present case, it is not the sharpest image that is to be found, but an image with the sharpness given by the standard stimulus. Therefore, it is not sufficient to determine only the position of an extremum of the criterion function, but the values themselves play an essential role. Moreover, the processes leading to blur also differ: In the case of the lens, blur can be understood as the result of the convolution of the image with a point spread function and affects the whole image in a similar way. In the case of gloss, in contrast, blur is confined to the projection of the object's surface, and the degree of blur at a surface point also generally depends on the orientation of the surface with respect to the observer. Despite these differences, the criteria proposed in the context of autofocus procedures for assessing relative image sharpness are also obvious candidates for the function h(r) we are looking for.

The methods proposed in the literature to address this technical problem can be broadly classified into three categories according to the type of information used (Ali et al., 2020; Groen et al., 1985; Krotkov, 1988): 1) spatial frequency-based methods that rely on the decrease of high-frequency image components with increasing blur, 2) methods that use statistics of the luminance histogram, such as the standard deviation to determine image contrast, and 3) feature-based methods that rely on statistics of local image features, such as the mean edge strength.

In studies comparing several methods (Ali et al., 2020; Groen et al., 1985; Jeon, Lee, & Paik, 2011; Jeon et al., 2011; Lee, Yoo, Kumar, & Kim, 2009; Shih, 2007), frequency- and feature-based statistics proved to be particularly reliable and robust against image noise. Therefore, one procedure from each of these two classes was selected and examined to what extent they meet the aforementioned quality criteria regarding the robustness of the roughness metric h(r) and the prediction of the subjects’ setting behavior. The first method is based on the discrete cosine transform (DCT), the second method on the gradient of an edge operator.

#### DCT procedure

The basic idea of the DCT procedure used here goes back to the autofocus method of Baina and Dublet (1995). The image for which the blur metric is to be computed is first converted to a grayscale image and then decomposed into 8×8 disjoint pixel blocks, for each of which a 2D DCT is performed. This results in 8×8 coefficients F(u,v) of the DCT per block:

$$\begin{array}{c} F\left(u,v\right)=\frac{c_{u}c_{v}}{4}\sum_{x=0}^{7}\sum_{y=0}^{7}p\left(x,y\right)g\left(x,u\right)g\left(y,v\right), \end{array}$$

where p(x,y) denotes the pixel value at position (x,y) in the block, the basis function g(α,β)=cos[(2α+1)πβ/16]. Furthermore $c_{\theta }=1/\sqrt{2}$ for θ=0 and $c_{\theta }=1$ otherwise.

The coefficient F(0,0) corresponds with the constant image component (DC). If we consider for F(i,j) the index vector (i,j), then its length encodes the frequency, its direction the orientation of the cosine gratings serving as basis functions, where F(i,0) corresponds with the horizontal, F(0,j) with the vertical direction.

For each block i, the value of the blur metric $U_{i}$ is calculated as the weighted average of the energy $F^{2}$ of all frequency components excluding the constant component:

(1) $$U_{i}=\sum_{u=0}^{7}\sum_{v=0}^{7}w_{u,v}F{\left(u,v\right)}^{2},$$

with $w_{0,0}=0$. In Baina and Dublet (1995), $w_{i,j}=1$ was assumed for all other weights. However, there are some findings, such as those from Jeon et al. (2011), that suggest that the robustness of the measure to image noise increases if the components are weighted differently or only subsets of the frequency components are included.

The value U for the entire image is then the arithmetic mean of $U_{i}$ over all relevant image blocks i. In the current application, only image blocks that lie completely within the outline of the object in the image are considered. This practice avoids a distorting influence of the (sharp) object edges.

If U(r|C) denotes the value of this metric for an image in which a surface with roughness r has been rendered under condition C, then the function we are looking for is h(r|C)≡U(r|C). For the experiment described in this article, there are 12 different conditions C resulting from combinations of three shapes, 2 illuminations, and 2 BRDFs. The value of r varies in 60 equidistant steps from r=0.001 to r=0.200.

#### Edge gradient (EG procedure)

Another proven method for determining image sharpness uses Sobel-type edge detectors (Krotkov, 1988; Shih, 2007). To this end, the image is converted to a grayscale image B and two 3×3 convolution kernels $G_{x}$ and $G_{y}$ are applied to determine the intensity gradients in the x and y directions. For the Scharr operator (Weickert & Scharr, 2002), which shows improved orientation independence compared with the Sobel operator, we have

$$\begin{array}{ccc} G_{x} & = & \frac{1}{32}\left[\begin{array}{ccc} 3 & 0 & -3\\ 10 & 0 & -10\\ 3 & 0 & -3 \end{array}\right],\\ G_{y} & = & \frac{1}{32}\left[\begin{array}{ccc} 3 & 10 & 3\\ 0 & 0 & 0\\ -3 & -10 & -3 \end{array}\right]. \end{array}$$

From these components, the magnitude of the gradient can be calculated as follows:

$$\begin{array}{c} U\left(x,y\right)=\sqrt{{\left[G_{x}*B\left(x,y\right)\right]}^{2}+{\left[G_{y}*B\left(x,y\right)\right]}^{2}} \end{array}$$

where * denotes the convolution operator.

The mean of U(x,y) within relevant image regions is then used as the roughness metric U. In the present case, the image region inside the outline of the imaged object is relevant. To decrease the effect of the object edge, the mask for the object region was made slightly smaller by an “erode” operation using a disk-shaped structural element with a diameter of 5 pixels.

Analogous to the DCT procedure, the functions h(r|c) are obtained by calculating this metric for surfaces with different roughness values and under different context conditions C.

### Test of the roughness metrics

Applying one of the procedures to the 720 stimuli used in the experiment yields 12 empirical curves h(r,C), each describing the roughness metric as a function of r for a given combination of BRDF, illumination, and shape. Smooth and strictly monotonically decreasing curves were obtained throughout, which could be fitted almost perfectly by equations of the form

(2) $$\hat{h}\left(x\right)=\alpha_{0}\exp\left(-\alpha_{1}^{2}x^{\alpha_{2}}\right)+\alpha_{3}x+\alpha_{4}.$$

All further investigations were carried out using this approximation and the (numerically determined) inverse function ${\hat{h}}^{-1}\left(x\right)$.

**Figure 11.**
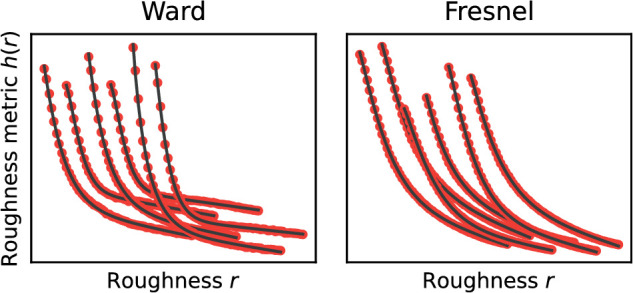
Values of the roughness metric h(r|C) calculated with the EG method (red dots) and the fits $\hat{h}\left(r|C\right)$ by the function given by Equation 2 (black line) for Ward- and Fresnel-BRDF. In the plots, the curves for the different conditions C are shown shifted horizontally with respect to each other by 0.03 in each case.

Figure 11 shows exemplary curves obtained with the EG method and the goodness of fit by the function $\hat{h}\left(x\right)$. The curves obtained with the DCT method look similar. However, the exact curve shape in this case also depends on the choice of the weights $w_{ij}$ for the individual frequency components in Equation 1.

#### Predicting the subjects’ settings

The obtained functions $\hat{h}\left(r,C\right)$ were used to predict the subjects’ mean settings following the logic shown in Figure 10. That is, for each trial, the roughness metric $I_{s}$ was obtained from $\hat{h}\left(r_{s}|S\right)$, where $r_{s}$ denotes the roughness value and S the condition combination used in the standard stimulus. The roughness setting predicted in the match is then $r_{p}={\hat{h}}^{-1}\left(I_{s}|M\right)$, where M denotes the combination of conditions realized in the match.

As a measure of the prediction error in trial i, $\Delta_{i}=\left(r_{p}-r_{m}\right)/s_{m}$ was chosen, where $r_{m}$ denotes the mean setting of the subjects and $s_{m}$ the associated SEM. The aggregate error across multiple trials $E=\sum_{i}\Delta_{i}^{2}$ was determined for the Ward-BRDF ($E_{W}$) and Fresnel-BRDF ($E_{F}$) separately across all associated trials. In these analyses, the 18 replicates of the same condition were considered as independent observations, resulting in a total of 288 mean values across 10 subjects.

#### Variants

A central question in the current investigation relates to the relative magnitude of $E_{W}$ and $E_{F}$. The robustness of the estimation is also of interest, that is, how these errors behave when boundary conditions are varied.

A first variation concerns the curves h(r|C). In the EG method, these are uniquely determined by the image (“fix”). However, the prediction of the setting by the described method is influenced not only by the shape of the curves, but also by the vertical position of the curves with respect to each other. To investigate the influence of this factor, a relative shift of the curves in y direction was allowed (“Shift”). Four variants were investigated for the DCT procedure. In condition “Fix,” it holds $w_{ij}=1$ (except for i=j=0), in the condition “Shift” additionally a vertical shift of the curves is allowed. In the condition “Freq,” any symmetric weight matrix w (with $w_{00}=0$) is permitted, and in the condition “All,” an additional vertical shift is also allowed. For each of the conditions “Shift,” “All,” and “Freq,” the free parameter values that minimized the errors $E_{x}$ were determined in an optimization procedure (more details can be found in Appendix A.1).

**Figure 12.**
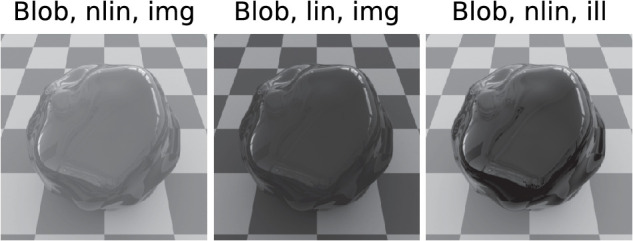
Illustration of the image variants used in the analysis. Left: Grayscale image of the stimulus with a gamma value of about γ=2.2 according to the sRGB standard. Middle: Linear grayscale image with γ=1.0, Right: Grayscale image (γ=2.2) of the isolated reflection. All stimuli are given for the Fresnel BRDF. Only the object region was included in the analysis, the background was masked.

As a further potential influence on the errors $E_{x}$, image properties were examined (cf. Figure 12). For this purpose, either the unmodified image (“Image”) or the mirror image rendered in isolation under identical conditions (“Ill”) were used as input. Because the isolated mirror image contains only the information relevant to the task, the comparison of these two conditions helps to determine how large the interfering influence of the diffuse component is. The corresponding grayscale images contained either the luminance information (“linear”), which was determined from the images based on the measured calibration data of the monitor used, or nonlinear grayscale values (“nonlinear”). In the latter case, the gray level was calculated as the linear combination L=0.2126*R+0.7152*G+0.0722*B of the color channels R,G,B.

### Results for the EG procedure

**Figure 13.**
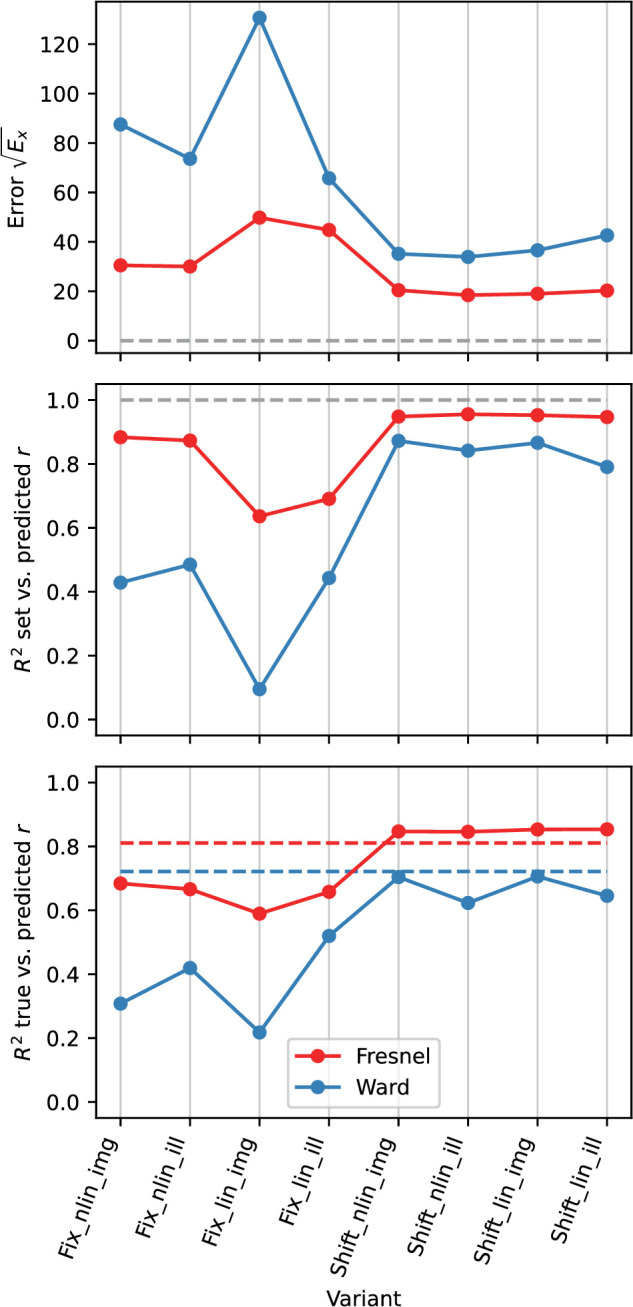
Comparison of the prediction errors with the EG procedure across different variants (luminance scale linear “lin” vs. non-linear “nlin”; diffuse component included “img” or excluded “ill”; fit method “fix” vs “shift”). Top: Root of the error $E_{x}$, middle: Determination coefficient $R^{2}$ for the comparison of observed $\left(r_{m}\right)$ and predicted $\left(r_{p}\right)$ roughness (cf. top panel in Figure 16). Bottom: Determination coefficient for the comparison of given $\left(r_{s}\right)$ and predicted $\left(r_{p}\right)$ roughness (cf. bottom panel in Figure 16). The horizontal dashed lines indicate the criterion for an optimal fit in each case. In the lower subgraph, these are the observed $R^{2}$ for the comparison of $r_{s}$ and $r_{m}$.

Figure 13 gives an overview of the prediction errors when using the EG procedure under the different variants tested. It can be seen that with the Fresnel-BRDF the prediction of settings was significantly better and varied also less across variants than with the Ward-BRDF.

However, a satisfactory prediction was only possible if a shift of the curves with respect to each other is allowed (variant “Shift”). The effect of the shift on the goodness of fit is particularly pronounced for the Ward-BRDF and also stronger with a linear luminance scale. Within the variant “Shift,” the prediction accuracy depends little on whether only the isolated mirror image or also the diffuse portion was included in the image. Surprisingly, with the Ward-BRDF the prediction quality tends to be lower with the isolated mirror image containing only the relevant information than with the diffuse component. The luminance scale has also only a small influence on the prediction quality, but there is a clear effect on the size of the shifts, which are about a factor of 2 larger with a linear scale.

**Figure 14.**
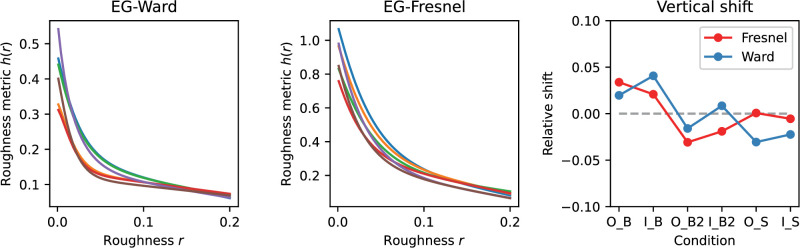
Shifted roughness metrics h(r|C) for the six illumination and shape combinations resulting from applying the EG procedure to variant “Shift_nlin_img.” The units are arbitrarily chosen to give “nice numbers,” so only relative values can be interpreted. The left and middle panel show the curves for the Ward- and Fresnel-BRDF, respectively. The fitted relative vertical displacements of the six curves are shown on the right. The shifts are given relative to the mean shift across the 6 curves for each BRDF (horizontal gray dashed line) in the same units as the curves. In the condition labels, “O” and “I” represent the two illuminations, “B,” “B2,” and “S” represent the three shapes.

In the following, the variant “Shift_nlin_img,” that is, with shift, nonlinear luminance scale and diffuse component included, will be considered in more detail. In this variant the errors are small and it presumably also describes the real situation most accurately. Figure 14 plots the curves of the roughness metric h(r|C) after the shift. The shapes of the curves, which are not affected by the shift, are more similar for the Fresnel BRDF than for the Ward BRDF. The degree of relative vertical displacement of the curves can be seen in the right panel in the figure. For the Ward-BRDF, the curves coincide after the shift at the lower end, that is, at large roughnesses, whereas for Fresnel-BRDF this convergence is less pronounced. The absolute values of the roughness metric for the Ward-BDRF are smaller overall (less than half as large) than for the Fresnel-BRDF. Although smaller values are to be expected given the weaker gloss impression with Ward-BRDF, the resulting difference seems to be exaggerated.

**Figure 15.**
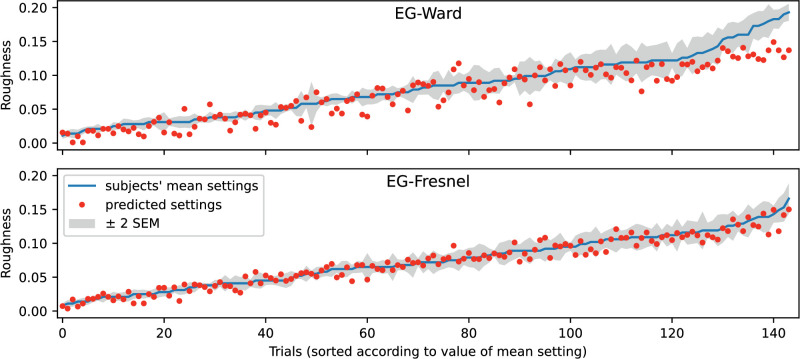
Comparison of the mean roughness settings ± 2 SEM and the predictions from the EG-procedure for Ward-BRDF (top) and Fresnel-BRDF (bottom). The predictions are for the variant “Shift_nlin_img.” The mean settings are given in ascending order.

Figure 15 shows the location of the predicted roughness values relative to the mean observed settings. Ideally, the prediction should lie within the error band shown in gray, which includes ± 2 SEM. Although this criterion is not consistently met, the predictions for the Fresnel-BRDF are at least not too far off. The fact that the prediction error is symmetrically distributed and, like the variance of the observed settings, tends to increase with increasing roughness, speaks for a good fit. In comparison, the fit for the Ward-BRDF is significantly worse. The scatter around the mean observed settings is larger and systematic deviations also occur, especially for larger roughness values.

**Figure 16.**
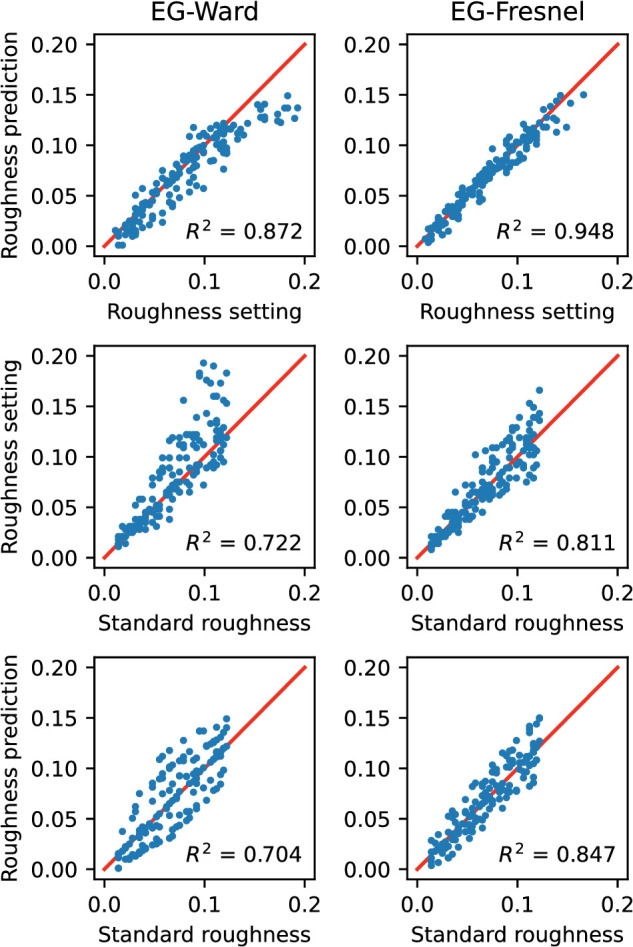
Comparison of observed values and model predictions for variant “Shift_nlin_img” using the EG procedure. Top: Predicted settings vs. mean observed settings. Middle: Standard roughness vs. mean roughness settings. Bottom: Standard roughness vs. predicted roughness setting.

Figure 16 presents further comparisons of observed and predicted values. The top graph shows the agreement of the prediction with the mean setting. As already shown in Figure 15, the model fit for the Fresnel-BRDF is significantly better than that for the Ward-BRDF. The middle graph shows the observed mean settings as a function of the target value in the standard and the bottom graph shows the corresponding distribution for the predicted values. Despite the relatively good agreement between the predicted and observed settings shown in the upper graph, systematic discrepancies are evident in the lower two plots. For both BRDFs, the increase in error with roughness is underestimated with respect to the true value: The prediction is less accurate for small roughness values and more accurate for large values than was the case for the subjects.

### Results for the DCT procedure

**Figure 17.**
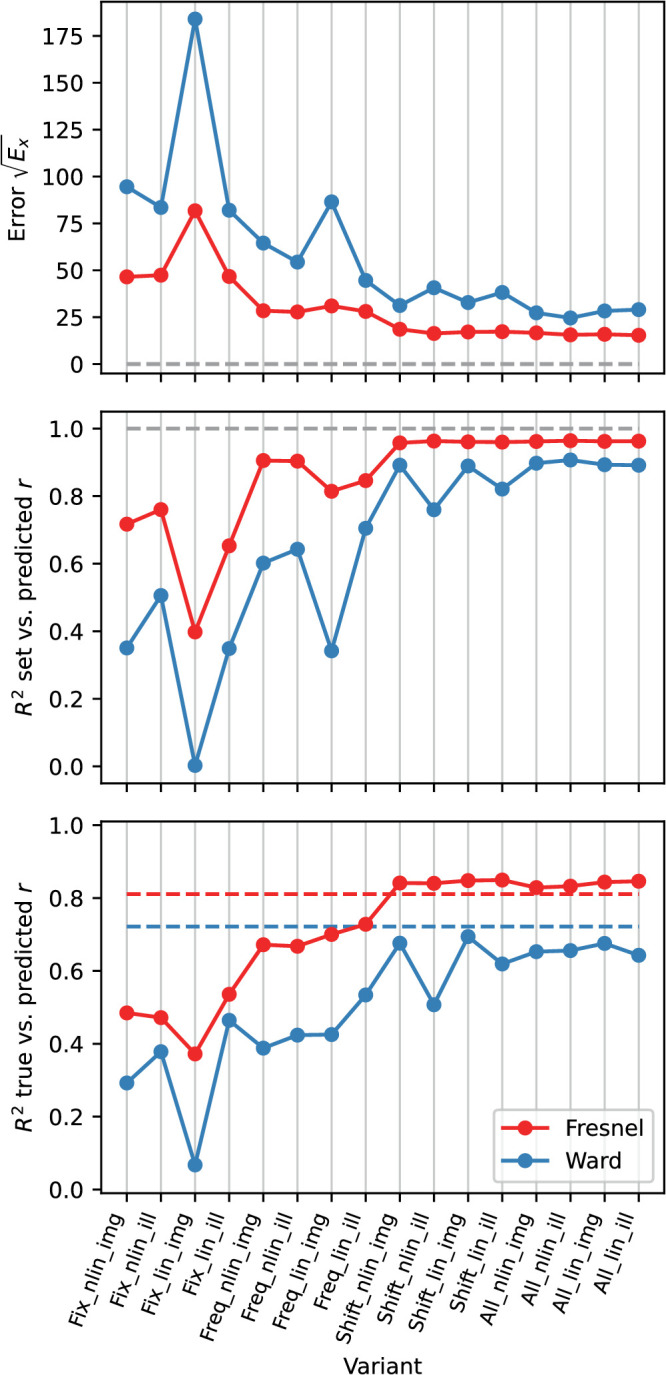
Comparison of the prediction errors with the DCT procedure across different variants (luminance scale linear “lin” vs. nonlinear “nlin”; diffuse component included “img” or excluded “ill”; fit method “fix,” “freq,” “shift,” and “all”). Top: Root of the error $E_{x}$. Middle: Determination coefficient $R^{2}$ for the comparison of observed $\left(r_{m}\right)$ and predicted $\left(r_{p}\right)$ roughness (cf. top panel in Figure 20). Bottom: Determination coefficient for the comparison of given $\left(r_{s}\right)$ and predicted $\left(r_{p}\right)$ roughness (cf. bottom panel in Figure 20). The horizontal dashed lines indicate the criterion for an optimal fit in each case. In the lower subgraph, these are the observed $R^{2}$ for the comparison of $r_{s}$ and $r_{m}$.

The results for the DCT procedure were evaluated analogously to those with the EG procedure. Figure 17 presents comparisons of the goodness of fit across the 16 variants tested. The “Fix” and “Shift” variants are directly comparable between the procedures, as are the variants with different luminance scales and with/without the diffuse component. In the “Freq” and “All” conditions, a different weight of the frequency components is also allowed, which affects the curve shape in each case.

It is clear that again allowing shifts of the curves relative to each other improves the prediction considerably. This finding is also true for the variant “Freq,” where the weights of the frequency components can be freely chosen. Conversely, the prediction in variant “Shift” is hardly worse than in variant “All.” The latter is especially true with the Fresnel-BRDF. However, it is again striking and unexpected that with the Ward-BRDF the prediction is partly better when the irrelevant diffuse component is included than with the isolated mirror image. With the Ward-BRDF, the different weighting of the frequency components seems to cause at least a somewhat greater stability of the estimation across different image variants (cf. Figure 17 top and middle panel). As with the EG procedure, the luminance scale has only a small influence on the prediction quality if curve shifts are allowed, but the size of the shifts are about a factor of two larger with a linear scale.

**Figure 18.**
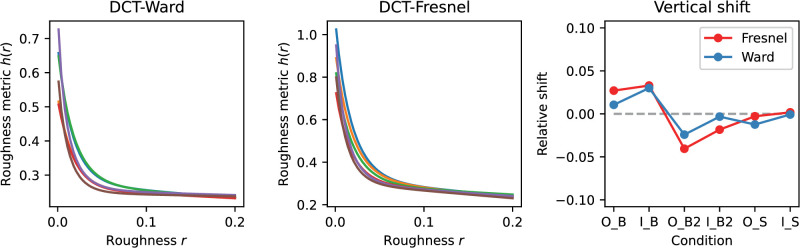
Shifted roughness metrics h(r|C) for the six illumination and shape combinations resulting from applying the DCT procedure to variant “All_nlin_img.” The left and middle panel show the curves for the Ward- and Fresnel-BRDFs, respectively. The fitted relative vertical displacements of the 6 curves are shown on the right. For more details, see Figure 14.

For the DCT method, the variant “All_lin_img” will be considered in more detail, since it provides the best prediction overall. Figure 18 shows the curves of the roughness metrics h(r|C) after the shift. The shapes of the curves for different context conditions C are again more similar for the Fresnel-BRDF than for the Ward-BRDF. However, the difference between the BRDFs seems smaller than for the EG procedure. For both BRDFs, the shifted curves coincide at large roughness values. Again, the values of the metric are lower overall for the Ward-BRDF than for the Fresnel-BRDF, but the difference is less pronounced and seems to be reasonably realistic if the subjects’ gloss ratings are taken as a reference.

**Figure 19.**
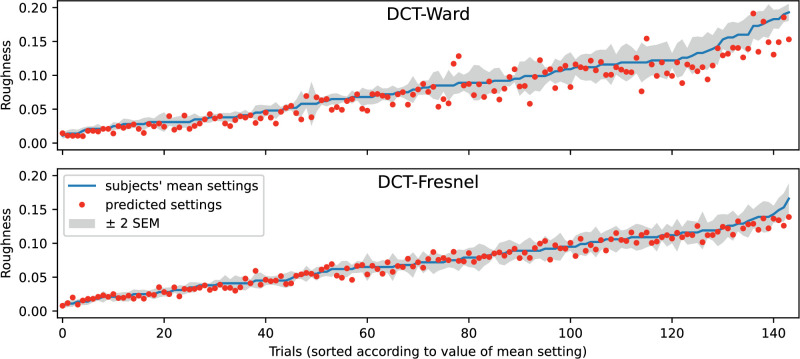
Comparison of the mean roughness settings ± 2 SEM and the predictions from the DCT procedure for Ward-BRDF (top) and Fresnel-BRDF (bottom). The predictions are for the variant “All_nlin_img.” The mean settings are given in ascending order.

Figure 19 depicts the location of the predicted roughness values relative to the mean settings of the subjects for the DCT procedure. The criterion that the prediction should lie within the error band shown in gray is again met to a good approximation for the Fresnel-BRDF. In contrast with the EG method, however, the fit here is also quite good for the Ward-BRDF, although systematic deviations can still be seen, especially for larger roughness values.

**Figure 20.**
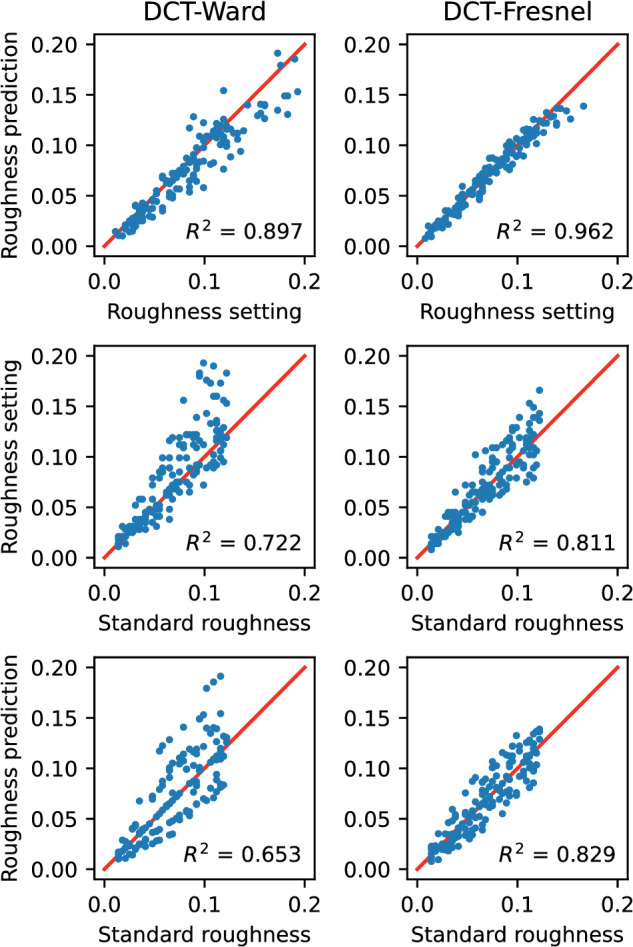
Comparison of observed values and model predictions for variant “All_nlin_img” using the DCT procedure. Top: Predicted settings vs. mean observed settings. Middle: Standard roughness vs. mean roughness settings. Bottom: Standard roughness vs. predicted roughness setting.

The more compact comparison of the agreement between the mean adjusted and predicted roughness for both BRDFs, shown in Figure 20, confirms the overall better fit of the data compared with the EG procedure. The pattern of deviations from the target value appears also slightly more similar in observation and prediction (middle vs bottom panels).

**Figure 21.**
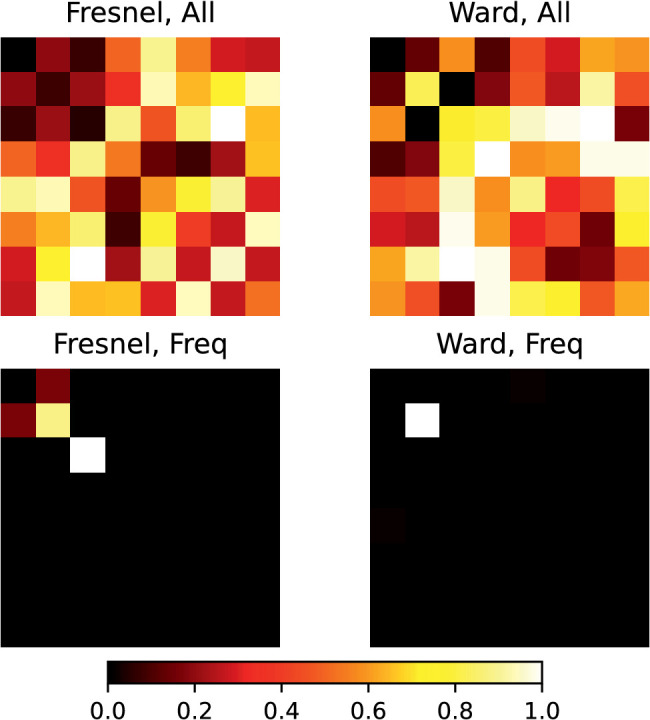
The weight matrix obtained in the DCT procedure for different fit methods (top: “All,” bottom: “Freq”) and the two BRDFs (left: Fresnel-BRDF, right: Ward BRDF). Because only the relative magnitude of the weights is critical, all weight matrices were normalized to a maximum of 1.

Figure 21 shows the symmetric weight matrices obtained for the two BRDFs in variants “Freq_nlin_img” and “All_nlin_img” where these parameters were determined within the optimization procedure. Without shifting the curves, that is, in variant “Freq,” the picture is very similar for both BRDFs, namely, that only very few frequency components make a substantial contribution. Although the resulting prediction is significantly worse than with shifted curves, it is at the same time significantly better than without adjusting the weights in variant “Fix.” This speaks at least for a different contribution of the individual frequency components.

If, in addition, a shift of the curves is allowed, the picture changes considerably and the weight pattern for the two BRDFs are also clearly distinguishable. In both cases there is a broad distribution of the weights. In the Ward BRDF, especially medium frequencies have a high weight, in the Fresnel BRDF, the distribution is more uniform and the contribution of high frequencies seems stronger. However, the exact distribution of the weights changed across the four subvariants, so that a detailed interpretation of the exact distributions does not seem to be appropriate.

### Discussion

This section addresses the goal of predicting the subjects’ settings from image features. There is a procedural analogy between the subjects’ task in the experiment and the goal of autofocus procedures used in cameras. This suggested using existing autofocus procedures as a guide for finding appropriate image features. In this technical application, methods relating either to edge gradients or to a frequency analysis of the image proved to be particularly promising. Two special cases from these procedural classes were adapted to the roughness estimation problem. The resulting EG and DCT procedures are relatively easy to apply and given their low complexity it does not seem impossible that they could be implemented in a similar form on the neural level.

To test how well the methods can be applied to the observed roughness settings, the stimuli used in the experiment were converted to grayscale images and the corresponding roughness metrics were calculated. In addition, this process was also done for three stimulus variants where the grayscale was changed and/or the specular component was isolated. In all cases, the roughness metrics were smooth functions of the roughness parameter α given in the BRDF, which could be approximated very well by a simple function with five free parameters. The shape of the curves was relatively robust to changes in the shape and illumination of the objects as well as across the just mentioned image variants. This robustness was even greater with the Fresnel-BRDF than with the Ward-BRDF.

In the experiment, roughness settings had been made across different illuminations and surface shapes. The degree of gloss constancy was reflected in the agreement between the roughness specified in the standard and the roughness set in the match. An image statistic, or a procedure based on it, is a suitable model of the underlying perceptual processes if the mean settings of the subjects and, in particular, the observed deviations from constancy are accurately reproduced.

In the verification of the model predictions, the relative vertical shifts of the roughness metric curves with respect to each other were allowed as essential free parameters. The results show that with a suitable choice of these five parameters per BRDF, the requirements for a proper metric outlined at the beginning of this section are very well met by both methods. The predictions for the Fresnel-BRDF are within or at least close to an error range of ±2 SEM around the subjects’ settings and the distribution around the “target value” is also reasonably symmetric. The prediction accuracy for the Ward-BRDF was somewhat lower. Here, systematic deviations occurred and the overall prediction error was larger. In a comparison of the two methods, the DCT method performed slightly better, if the weights of Frequency components were treated as free parameters (variant “All”). However, considering that this involved adjusting 35 additional free parameters per BRDF, the improvement seems rather small. If instead the “Shift” variant of the DCT procedure was chosen, almost identical results were observed with both procedures.

A possible explanation for the dependence of the prediction accuracy on the BRDF could be that with the Ward-BRDF the subjects did less consider only the static image contents, but resorted more to adjustment strategies or also used the stimulus changes during the adjustment as a criterion, which of course cannot be reproduced with the methods considered here. This interpretation is also supported by the unexpected asymmetry of the errors observed with the Ward-BRDF when swapping the illuminations of standard and match.

#### Interpretation of the roughness metrics

The roughness metrics are understood as low-level cues for the roughness of the surface, which can be determined directly from the input signal. As shown in Figure 10, it is assumed that the curves describing the roughness metric as a function of surface roughness, directly determine the subjects’ adjustment behavior and thus the degree of constancy.

The degree of constancy is primarily determined by the shape of the curves, with maximum constancy occurring when the curves match completely. The finding that with both procedures the curves determined for the Ward-BRDF vary more in their shape than those for the Fresnel-BRDF is consistent with the overall lower constancy found in the experiment with the Ward-BRDF. In this sense, the procedures provide objective evidence that the physically plausible reflections produced using a Fresnel-BRDF can lead to better constancy performance than BRDFs that do not correctly reproduce Fresnel effects (Faul, 2019).

##### The role of vertical shifts

When checking the procedures, vertical shifts of the curves with respect to each other were allowed to eliminate the possible influence of an incorrect determination of the zero point, so that only the shape and the range of values of the curves mattered. In absolute terms, the size of the shifts was relatively small, but the nature of the assumed matching processes implies a high sensitivity of matching errors to deviations from the correct vertical alignment of the curves.

A possible interpretation of these shifts is that they reflect inadequacies of the specific models and would not be necessary if the correct image statistics were used. Alternatively, adaptation may play a role: The fact that the shifts needed for the best prediction turned out to be considerably smaller with a nonlinear than with a linear luminance scale points to the possibility that a nonlinear compression of the scale owing to brightness adaptation, which has been ignored in the analysis, may have a considerable influence on the shifts.

According to the present results, the shifts have the effect that the curves are aligned at large roughness values. This finding suggests as a further possibility that the zero points of the scales are determined in a kind of self-calibration process. But how can the zero point be determined for a single observation when only one point of the curve is known, so to speak? Essentially, this requires knowing the response of the responsible mechanism given a fully diffuse surface (under the same conditions) and this “zero response” must then be subtracted in each case to obtain a standardized response. This “zero response” could be estimated in several ways. One is by reference to a point in the external world at which the mechanism produces a minimal response, another is by reference to internal sources, for example, low-frequency components in a frequency analysis of the input, blurred image content in the parafovea, or the blurred images produced during lens modifications to focus the retinal image.

##### Prediction of errors

The negatively accelerated curves resulting from the EG and DCT procedures can plausibly explain the increase in error observed in the experiment for large roughness values if one assumes a constant just noticeable difference in the roughness metric: To reach the just noticeable difference, greater changes in roughness are required for large roughness values than for small ones. Thus, the difference threshold for surface roughness should be larger for large roughnesses.

##### Perceived roughness

For the prediction of the subjects’ settings it is sufficient to know the curves h(r|C). However, these presumably do not directly determine the perceived roughness, which seems to have an approximately linear relationship to the varied roughness parameter α of the BRDFs (see Pellacini et al., 2000, and the results of the current experiment). To achieve this with the given roughness metrics h(r|C), the function f postulated in Figure 10, which maps the roughness metric into perceived roughness, must approximately correspond to the inverse function $h^{-1}\left(r|C\right)$. However, it must be assumed that f is not context-specific, but refers to an average function h(r), because only then does the perceived roughness also depend on context effects. If one assumes that the gloss ratings in the experiment reflect perceived roughness, then the findings shown in Figure 5 are consistent with this expectation.

##### Relevant information

Despite certain differences in the curves obtained with the EG and DCT procedures, a high degree of agreement can be seen overall. This is also reflected in similar predictions. This outcome suggests that both methods exploit similar information. Both can be taken as statistics of local variability in a narrowly defined image region. Specifically, for the EG procedure this is the luminance gradient in a 3 × 3 neighborhood, while for the DCT procedure it is the local variance in an 8 × 8 neighborhood (Baina & Dublet, 1995).

##### General criteria for suitable metrics

In the search for alternative statistics, some criteria emerged that a viable method must meet. First, it seems to be of crucial importance that the object edge itself is not included. Because of the problem of edge effects, global methods, such as the power spectrum of the discrete Fourier transform of the masked object, seem less suitable. Another important property seems to be that the metric depends continuously on the image content. For this reason, methods that use thresholds, such as edge detection methods like the Canny operator, are problematic. In informal studies with the stimuli used here, such methods did not yield smooth curves. It also seems important to consider spatially constrained neighborhoods, otherwise local effects of image sharpness are confounded with global stimulus properties. For this reason, criteria based on luminance histogram statistics are probably less reliable (Ali et al., 2020).

#### Extensions

In the current investigation, the test of further procedures was omitted, because already the tested procedures allow an almost perfect prediction of the experimental data. A further test of this and alternative procedures should preferably be carried out on the basis of data covering a wider range of context conditions, for example, with regard to shapes, illuminations, surface texture and the nature of neighboring objects.

An important criterion that should be examined when considering other methods described in the autofocus literature is whether they can be generalized in an obvious way to a spatially limited region of blur.

The tested methods share with many other proposed methods that the roughness metric is a simple sum statistic, usually the mean, of the distribution of the response of local feature detectors. However, other properties of these distributions could also be relevant, e.g., skewness, which in the context of the luminance histogram has been ascribed a relationship to gloss impression (Motoyoshi, Nishida, Sharan, & Adelson, 2007).

A possible extension could also be to weight the local statistics with the strength of reflectance and thus emphasize the contribution of information in regions where the intensity of the mirror image is high. Provided the shape of the object is known, object regions of high reflectance can be determined from the Fresnel equations or a simplified version of the relationship between orientation and strength of reflection.

### Crucial differences between Fresnel- and Ward-BRDFs

Significant differences between the Ward- and Fresnel-BRDFs have emerged, both in the analysis of the empirical data, and in the evaluation of potentially relevant image statistics. This finding is somewhat surprising; in both cases, surface roughness is similarly mapped into the degree of blurring of the mirror image. An important question, therefore, is what causes these differences. One possibility would be that there are nevertheless small differences in blur, another that the two BRDFs weight the mirrored regions of the environment differently.

**Figure 22.**
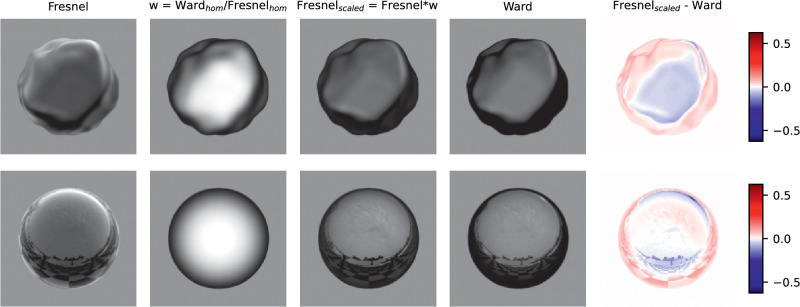
Rescaling of the isolated mirror image obtained with the Fresnel-BRDF to optimize the similarity with the mirror image calculated with the Ward-BRDF. From left to right: 1) initial image, 2) the local rescaling factor w is obtained as the quotient of the specular reflections of a homogeneous illumination in the object obtained with Ward- and Fresnel-BRDF, respectively, 3) scaled Fresnel reflection, 4) reflection with Ward-BRDF under the same condition, and 5) difference between scaled Fresnel reflection and reflection with Ward-BRDF.

To explore this question, the isolated mirror image computed with the Fresnel BRDF was scaled in such a way that the weighting of the different spatial directions is similar to that of the Ward BRDF (cf. Figure 22). To this end, all objects were rendered under homogeneous illumination and without a floor. The quotient of the mirror images generated with the two BRDFs gave the relevant weighting factor w (see second column in Figure 22). The scaled image is quite similar to the mirror image observed with the Ward-BRDF (see third and fourth column). However, as the difference images in the last column shows, residuals of the original mirror images remain. The blue portions in the difference image indicate where the “Ward mirror image” is brighter than the scaled “Fresnel mirror image,” the red portions the reverse.

The difference image shows very clearly that different parts of the environment are imaged with high intensity when using the Fresnel-BRDF instead of the Ward-BRDF. The high reflectance that is present at the edge of the objects when using the Fresnel-BRDF means that in the tested scene the structured floor is clearly imaged regardless of the illumination, which could have contributed to the high constancy regardless of shape and illumination.

**Figure 23.**
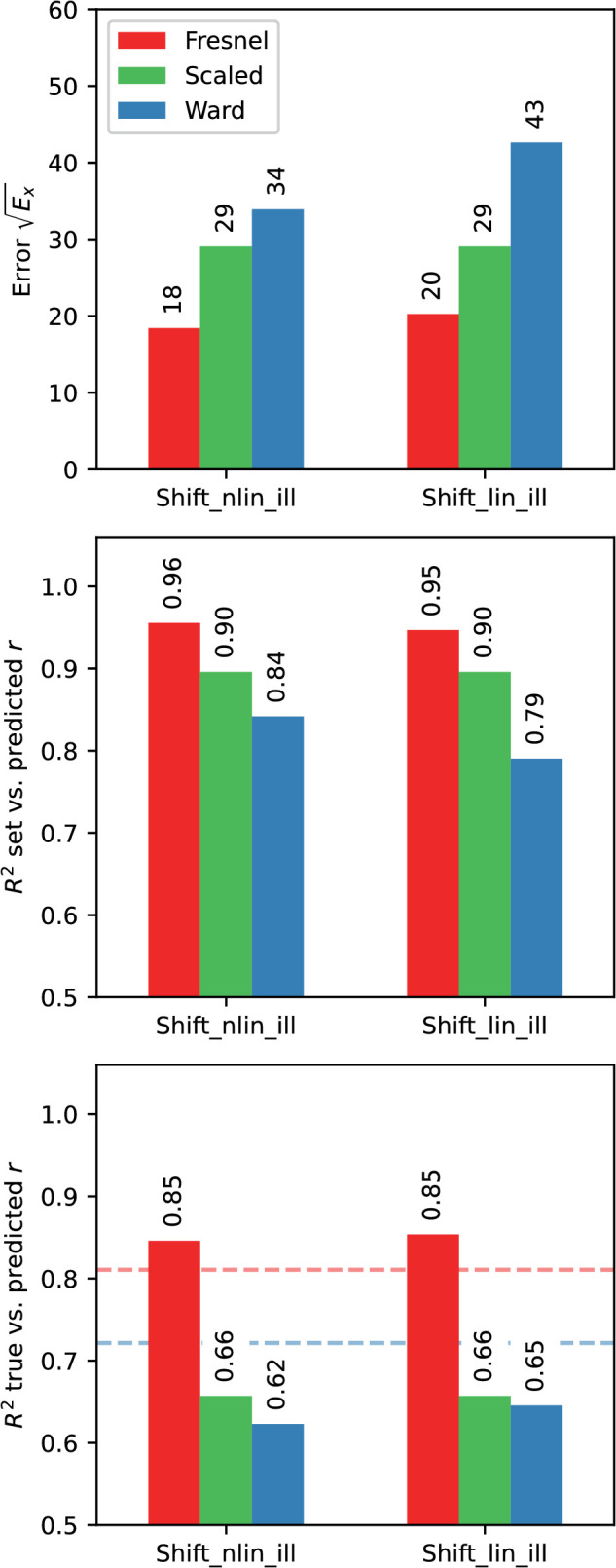
Comparison of error and prediction quality for the scaled Fresnel reflection compared to the specular reflection determined with Ward or Fresnel BRDF. More information on the depicted indices can be found in the caption of Figure 13.

Figure 23 compares for the EG procedure the predictions of this scaled version of the mirror image with those based on the isolated mirror images determined with the Ward- and Fresnel-BRDFs. It can be seen that with the scaled version, the deviations from the subjects’ settings are between that of the Ward and the Fresnel-BRDF. This suggests that the different weighting of spatial directions is indeed responsible for a part of the difference between the Ward- and Fresnel-BRDFs. In addition, however, more complex properties of the mirror image, such as those resulting from interactions of light with the scene, for example, shadowing of indirect light sources and an uneven distribution of surface reflectances in the scene, also seems to play a role. These are essentially responsible for the fact that the difference images of scaled Fresnel- and Ward-BRDFs are not zero.

## General discussion

The results of experiment and modeling suggest that the perceived roughness of a surface and the degree of constancy of the roughness impression across changes in its shape and illumination may depend on the reflection model used. In the situation studied, a BRDF in which Fresnel effects are correctly reproduced was found to be advantageous. That is, the gloss impression varied less and the constancy was greater. To capture and quantify contextual influences on the gloss impression in real scenes, it therefore seems necessary to use BRDFs in which Fresnel effects are correctly reproduced.

At least in the situations simulated here, the subjects’ settings could be predicted very well by simple image statistics related to the local variance of the mirror image. This finding is remarkable, given that the simple matching model employed here ignores adaptation processes, adjustment strategies, the influence of other gloss cues and the use of dynamic information during matching. The predictions were also quite robust to variations in the image material, for example, to a change in the luminance scale or to variation in the diffuse part of the reflection. The modeling also correctly reproduces the observed differences between Fresnel- and Ward-BRDFs, correctly predicts the increase in error with increasing standard roughness, and the procedures also appear simple enough to be physiologically realizable.

The similarity of the predictions of the two procedures compared indicate that the local variance in image regions within the object, which enters into both procedures, plays a significant role in gloss impression. There are, of course, a number of alternative methods that can be used to capture this information. A detailed comparison of their relative advantages or disadvantages can only be made on the basis of more extensive stimulus material.

### Limitations of the modeling and relation to other gloss cues

The “cues” considered in the modeling were studied and validated under highly restricted conditions. In everyday situations, it is likely that they are informative only in combination with other cues that already indicate the presence of specular reflection. This relates to the important distinction between the assignment of a stimulus to a certain class and the determination of specific properties of the exemplar. The second step is usually conditional on the first and may draw on different or additional information and cues. One reason for this is that the second step generally relates to property dimensions that are class specific. For example, trying to determine the transmittance properties of a material is only meaningful if it is already classified as light transmitting.

Thus, although the hypothesized image statistics provide new insights into how specular roughness might be computed directly from images, these statistics should only be used where the position of specular reflections are already known by some other means. Within such regions, the proposed statistics seem to provide reliable information on the degree of surface roughness. In the context of a broader model that takes multiple cues into account, they might be understood as a concretization of the gloss criterion of edge sharpness that is frequently encountered in the literature (Marlow & Anderson, 2013; Marlow et al., 2012).

With respect to the question how region of specular reflection could be determined in static images, previous work has argued that specular reflections have a characteristic orientation and position with respect to smooth shading gradients generated by the diffuse component of reflectance (Marlow et al., 2011; Kim et al., 2011). Specifically, they argued that specular reflections share the same orientation as diffuse shading gradients (i.e., exhibit “orientation congruence”), and the position of specular highlights is generically located closer to intensity maxima of diffuse shading than intensity minima (i.e., exhibit “position congruence” with respect to diffuse shading). However, these analyses ignored the impact of Fresnel effects on specular image structure. Future work should assess whether these two congruencies between specular reflections and diffuse shading generalize to other BRDFs in which Fresnel effects are correctly simulated. The present results show that there are substantial differences in the position of specular reflections between the Ward- and Fresnel-correct BRDFs, which raises questions about the generalizability of the concept of position congruence in particular.

The image statistics proposed herein are blind to the 3D shape of a glossy surface, but a growing body of work indicates that various aspects of material perception including gloss are computed at the level of 3D shape representation. Specifically, it has been shown that identical image gradients can appear as either specular reflections or as diffuse shading gradients depending on an image's 3D shape interpretation (Marlow & Anderson, 2015, 2016; Marlow, Todorović, & Anderson, 2015). One interpretation of these effects is that the perception of specular roughness may not just depend on the 2D image statistics proposed herein, but also on the apparent 3D orientation of highlights and apparent rate of surface curvature across them. Alternatively, considering the distinction between classification and property estimation, one could also argue that these findings relate more to the (logically) preceding step of classifying image regions as areas of specular reflection and that they are less relevant for the subsequent characterization of the properties of the specular reflection and the corresponding surface. Although only indirectly related to this specific question, it may be of interest to note that the systematic differences between Fresnel- and Ward-BRDFs found in the present study suggests an *implicit* influence of 3D shape on roughness estimates via the spatially varying reflection strength that, owing to Fresnel effects, depends on 3D shape.

In a sense, the present approach follows the common practice to consider image statistics as predictors of material properties, including gloss (Motoyoshi & Matoba, 2012; Nishida, 2019; Nishida & Shinya, 1998; Sawayama & Nishida, 2018; Sharan, Li, Motoyoshi, Nishida, & Adelson, 2008; Wiebel, Toscani, & Gegenfurtner, 2015). The proposed statistics are usually related to histograms of the intensities in the raw image (Motoyoshi et al., 2007) or in images belonging to a subband analysis (Motoyoshi & Matoba, 2012; Sharan et al., 2008). For example, to predict surface albedo Sharan et al. (2008) consider statistics relating to edges in the image that are similar to the ones proposed here. In contrast with the statistics of raw image intensities, such measures are sensitive to image structure and also take into account that the raw image properties are not available to the visual system. In such approaches, the relevant statistics are typically derived from rather vague deliberations regarding their relation to the target property and often several statistics are combined. The present statistics, in contrast, are more specific. For one, because they are concerned with a very specific property of glossy surfaces and also because they explicitly try to capture the blurriness of the mirror image, which is well-known to be a direct consequence of surface roughness. In line with this, it was found that the proposed image statistics (if applied to glossy surfaces) provide very precise and reliable information on the degree of surface roughness and also accurately predict the performance of subjects in a roughness matching task.

The present results indicate that using either the Fresnel- or the Ward-BRDFs can lead to *different* predictions with respect to the constancy of the perceived roughness of glossy surfaces. However, one cannot conclude that the Fresnel-BRDF is always *beneficial* for the gloss impression and the constancy of the perceived roughness across viewing conditions. For example, including a textured floor in the scenes may have been favorable for the Fresnel-BRDF because it resulted in a comparatively constant influence near the edge of the object, where there is strong reflection owing to Fresnel effects, which may have stabilized the sharpness estimate. Owing to the limited number of conditions that could be tested, the extent to which the proposed image statistics are specific to certain contextual conditions, for example, certain shapes or illumination types, remains unclear. To explore their general usefulness, further studies with more extensive stimulus material are necessary.

## Conclusions

The current findings indicate that the constancy of perceived roughness of glossy surfaces across different illuminations and shapes can strongly depend on the BRDF used. Improved gloss constancy with correctly simulated Fresnel effects was not only observed in subjective gloss matches but also in two image statistics proposed as potential cues for surface roughness.

These results complement those presented in Faul (2019) on the influence of Fresnel effects on the gloss impression and the constancy of perceived gloss strength. Taken together, they strongly suggest that Fresnel effects have a significant impact and should be properly simulated in empirical studies on gloss perception.

## Figures and Tables

**Figure 24.**
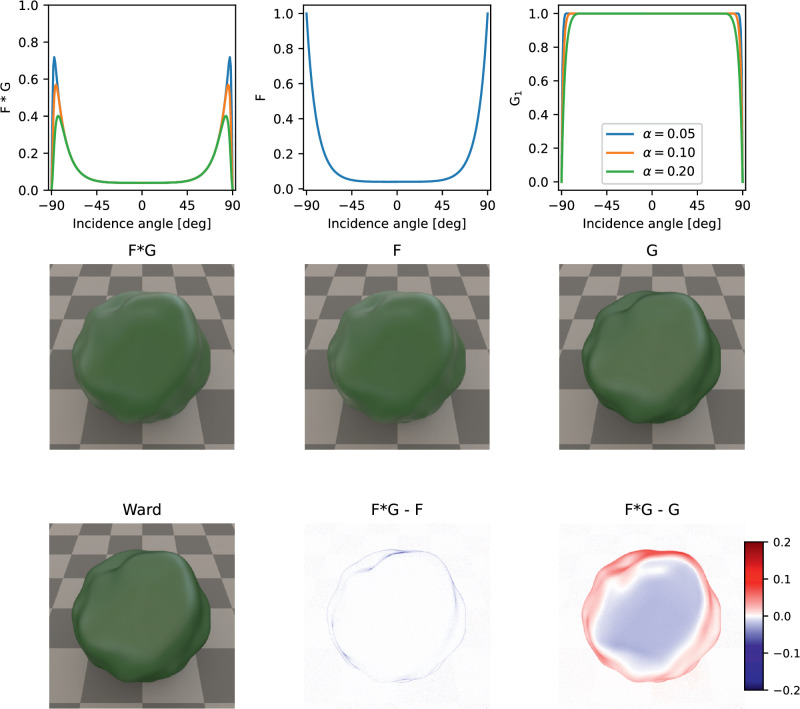
Influence of Fresnel reflection F and geometric attenuation factor G on the rendering in the Fresnel-BRDF. Top row from left to right: The combined effect of G and F, the Fresnel reflection F and the function $G_{1}$. Middle row from left to right: Correct rendering with F and G, isolated F effect, and isolated G effect. Bottom row: The left panel shows the rendering with the Ward-BRDF. The middle and right panel show the luminance difference between the full rendering and renderings with the isolated factor show directly above. The refractive index was 1.5 and the roughness α=0.15. The renderings are shown with a gamma of 2.2, the difference images are linear. See text for details.

## Appendix

### Details on the fit procedures used in the modeling

The fits performed in the modeling of the experimental data used procedures from the Python module scipy.minimize. To fit the approximating function $\hat{h}\left(r\right)$ to the roughness metric values resulting from the EG and DCT procedures, the Minimize method from the “lmfit” front-end to scipy.minimize was used. To find the shift and/or weight parameters that minimize $E_{x}$, the Python procedure scipy.minimize with the method “L-BFGS-B” was used. In variants of the DCT procedure, in which the weights $w_{ij}$ were free parameters, nested fits of the functions h(r,C) depending on these parameters, were necessary. To enhance the robustness of the fits, 10 repetitions of the procedure with randomized starting values were performed and the version with the lowest value of $E_{x}$ was selected.

### The relative influence of Fresnel effects and geometric attenuation on reflection strength

The Ward-BRDF does not only ignore Fresnel effects, but also a geometric attenuation factor: The Gaussian distribution has shown up repeatedly in theoretical formulations of reflectance [...], and it arises from certain minimal assumptions about the statistics of a surface height function. It is usually preceded by a Fresnel coefficient and geometrical attenuation factors, and often by an arbitrary constant. Since the geometric attenuation factors are typically difficult to integrate and tend to counteract the Fresnel factor anyway, we have replaced all of these coefficients with a single normalization factor that simply insures the distribution will integrate easily and predictably over the hemisphere Ward (1992, p. 268).

Thus, in a strict sense, using the Fresnel-BRDF proposed by Walter et al. (2007) does not isolate the contribution of Fresnel effects F, but the difference to the Ward-BRDF is also due to the inclusion of the attenuation factor G. The attenuation factor leads to a darkening of the specular reflection near grazing angles and thus counteracts Fresnel effects, which are reflected in an *increase* of specular reflection near grazing angles. This means that an increase of reflection strength near grazing angles above that observed with the Ward-BRDF can only be due to F.

In the BRDF, F, G, and the Beckmann distribution D are multiplied with each other. The upper row of Figure 24 illustrates the effects of F and G. $G(\omega_{i},\omega_{o},m)$ is a function of the incidence direction $\omega_{i}$, the reflection direction $\omega_{o}$ and the local surface normal m. $F(\omega_{i},m)$ is a function of $\omega_{i}$ and m. The function G is separable: $G\left(\omega_{i},\omega_{o},m\right)\approx G_{1}\left(\omega_{i},m\right)G_{1}\left(\omega_{o},m\right)$. The top right panel in Figure 24 shows the rational approximation to $G_{1}$ for three realistic roughness values (compare Figure 8 and Eq. 27 in Walter et al., 2007). That is, the attenuation works only near grazing angles. The middle panel in Figure 24 shows F for a typical refractive index of 1.5. Finally, the top left panel shows the combined effect of G and F for $\omega_{o}=\omega_{i}$: G attenuates F near grazing angles and this effect increases with α.

The middle row of Figure 24 shows renderings with a patched version of the Mitsuba renderer, in which either the effect of G or F was selectively discarded (and thus the other factor isolated). To eliminate Fresnel effects, F was set to a constant value $f=2{\left(ior-1\right)}^{2}/{\left(ior+1\right)}^{2}$, i.e., to twice the value for perpendicular incidence with a given refractive index ior. To eliminate G, this factor was was set to 1. The renderings are for ior=1.5 and α=0.15. A comparison of the correct rendering with the restricted renderings, which isolate F and G, respectively, reveals that the effect of G is very small. As expected, the reflection strength without attenuation G is slightly increased near grazing angles. When only the factor G is considered, the result is very similar to that obtained with the Ward-BRDF. The difference in the relative effects of isolating F and G can also be seen in the difference images that compare the luminance of full and restricted renderings.

Together, these findings indicate that, at least for the parameter values realized in the experiment, the differences between the stimuli obtained with Fresnel- and Ward-BRDF are almost completely due to the inclusion of Fresnel effects.
